# Overweight/Obesity and Hyperinsulinemia Impair Oocyte Developmental Competence via Cumulus Cell Dysfunction and Oxidative Stress-Mediated Follicular Microenvironment Remodeling

**DOI:** 10.3390/ijms27188246

**Published:** 2026-09-16

**Authors:** Ting Wu, Yuxiao Zhou, Guangxin Yao, Ying Ding, Shuanggang Hu, Zhide Ding

**Affiliations:** 1 Department of Reproductive Medicine, Shanghai Key Laboratory for Assisted Reproduction and Reproductive Genetics, Ren Ji Hospital, Shanghai Jiao Tong University School of Medicine, Shanghai 200135, China; 2 Department of Histology, Embryology, Genetics and Developmental Biology, Shanghai Key Laboratory for Reproductive Medicine, Shanghai Jiao Tong University School of Medicine, Shanghai 201318, China

**Keywords:** overweight/obesity, hyperinsulinemia, cumulus cells, in vitro maturation, immune activation, N-acetyl-L-cysteine (NAC)

## Abstract

Overweight and obesity, frequently accompanied by hyperinsulinemia, are established risk factors for female infertility. Cumulus cells (CCs) are hypothesized to play a crucial role in this etiology, yet the underlying mechanisms remain largely elusive. In the current study, we investigated the impact of hyperinsulinemic overweight/obesity on the follicular niche using integrated clinical analysis and in vitro maturation (IVM) assays. Follicular fluid and CCs from patients stratified by body mass index (BMI) and fasting insulin levels were evaluated. Immature oocytes, including oocytes at the metaphase I (MI) and germinal vesicle (GV) stages, underwent IVM using conditioned media or co-culture with autologous or allogeneic CCs. Additionally, CC transcriptomic profiling was performed using RNA-seq. We found that overweight/obesity, particularly when accompanied by hyperinsulinemia, induced CC immune activation, characterized by elevated follicular IgG, IgM, and pro-inflammatory cytokines. This pathological niche significantly compromised oocyte maturation. Notably, co-culture with CCs from normal-weight patients or supplementation with the antioxidant N-acetyl-L-cysteine (NAC) effectively reversed these defects. In conclusion, hyperinsulinemic obesity impairs oocyte competence through CC dysfunction and follicular remodeling. Restoring the CC-oocyte microenvironment via co-culture with healthy CCs or antioxidant therapy represents a promising strategy to alleviate this impairment and improve IVM efficiency in clinical practice.

## 1. Introduction

Granulosa cells (GCs) including cumulus cells (CCs) and mural granulosa cells are integral to the follicular microenvironment, serving as essential regulators of oocyte meiotic maturation, cytoplasmic remodeling, and developmental competence [1,2,3,4]. Given the oocyte’s limited intrinsic antioxidant capacity, GCs also preserve redox homeostasis, shielding the oocyte from oxidative damage and lipotoxicity, which is crucial to oocyte quality [5]. Conversely, the lack of GC support during in vitro maturation (IVM) leads to aberrant nuclear and cytoplasmic maturation, along with compromised meiotic progression [6]. Notably, restoring the cumulus-oocyte complexes (COCs) via co-culture re-establishes this supportive microenvironment, effectively reversing these maturation defects [7,8]. Therefore, cumulus cells serve as essential, active regulators of oocyte maturation in both physiological and in vitro systems.

Over the past few decades, obesity increasingly threatens female reproductive health, conferring an approximately 60% higher risk of infertility compared with normal-weight women [9]. Excessive adiposity drives hyperinsulinemia, systemic inflammation, and oxidative stress, which converge to alter the follicular microenvironment [10,11]. Elevated BMI is consistently associated with profound changes in follicular fluid composition, including increased levels of free fatty acids [12], insulin [13], C-reactive protein [14], leptin [10,15] and pro-inflammatory cytokines [16]. These perturbations correlate strongly with impaired oocyte developmental competence [13,17], as evidenced by increased spindle defects, chromosomal misalignment, and reduced maturation rates in both animal models [18] and clinical IVM cycles [19].

Although these findings imply that obesity reshapes the follicular niche, emerging evidence suggests that intrinsic granulosa cell dysfunction underlies many of these alterations [17]. Proteomic and transcriptomic studies reveal that obesity induces mitochondrial dysfunction, disrupted redox balance, and impaired fatty acid oxidation capacity in cumulus cells [20]. Such dysfunction is likely to disrupt cumulus cell–oocyte communication, thereby compromising oocyte quality [21].

Nevertheless, how obesity-associated factors (e.g., free fatty acids) and hyper-insulinemia directly impair human cumulus cell function and disrupt the follicular microenvironment remains unclear. This study therefore examined whether hyperinsulinemia drives cumulus cell dysfunction and oocyte maturation failure. Based on these insights, we further assessed whether targeted interventions in the IVM microenvironment—via healthy cumulus cell co-culture or antioxidant N-acetyl-L-cysteine (NAC) supplementation—could restore oocyte competence under hyperinsulinemic and obese conditions, providing a practical approach to enhance assisted reproductive outcomes.

## 2. Results

### 2.1. BMI-Dependent Modulation of Oocyte Maturation by Cumulus Cells In Vitro

**Figure 1:**
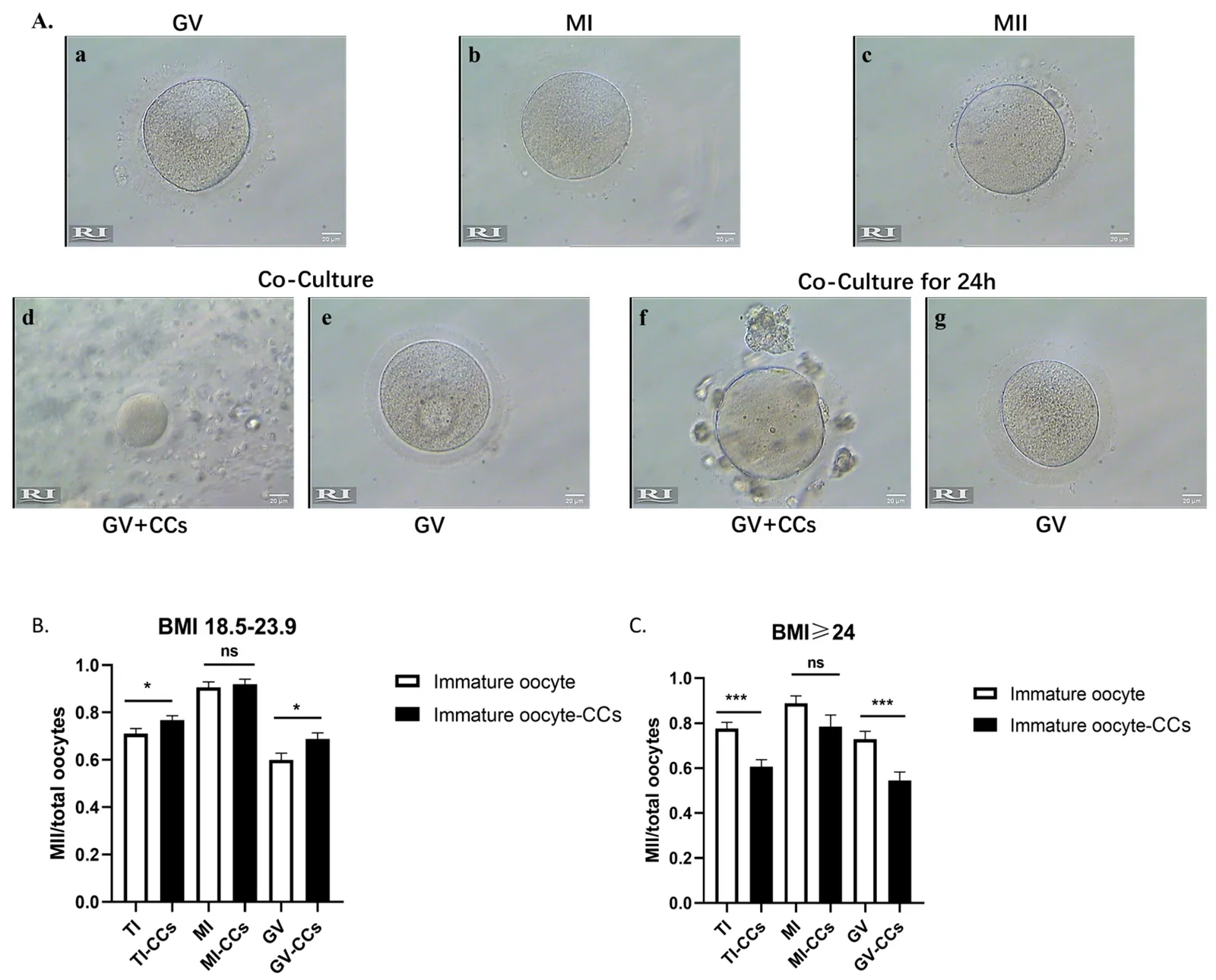
Impact of autologous cumulus cells (CCs) from different BMI groups on in vitro oocyte maturation. (**A**) Representative bright-field images of oocytes at the GV (**a**), MI (**b**), and MII (**c**) stages. (**d**–**g**) Representative images of co-culture and control groups. CCs are shown adhering to GV oocytes at 0 h (d) and 24 h (f), whereas control oocytes lack surrounding CCs at corresponding time points (e,g). Scale bar: 20 µm. (**B**,**C**) Maturation rates of oocytes co-cultured with CCs from normal-weight (18.5 ≤ BMI < 24.0 kg/m^2^, (**B**)) and overweight/obese (BMI ≥ 24 kg/m^2^, (**C**)) groups. Data are presented as mean ± SEM; Statistical significance: ns, not significant, * *p* < 0.05, *** *p* < 0.001. Sample sizes: Normal-weight (MI: *n* = 139, *N* = 175; MI-CCs: *n* = 143, *N* = 178; GV: *n* = 248, *N* = 305; GV-CCs: *n* = 292, *N* = 359); Overweight/obese group (MI: *n* = 58, *N* = 102; MI-CCs: *n* = 55, *N* = 95; GV: *n* = 135, *N* = 149; GV-CCs: *n* = 161, *N* = 189). Note: TI, total immature oocytes (MI + GV); *n*, number of independent patients; *N*, total number of oocytes.

Immature oocytes were cultured in G_1_ medium under controlled conditions (37 °C, 5% O_2_, 6% CO_2_, balanced with N_2_) using two systems: (1) an oocyte-only control group (control) and (2) a co-culture group with autologous cumulus cells (CCs). As shown in Figure 1(Aa–Ac), representative oocytes at the GV, MI, and MII stages are illustrated. CCs remained clinging around oocytes throughout the 24 h culture period (Figure 1(Ad,Af)), whereas control oocytes lacked surrounding CCs at both 0 h and 24 h (Figure 1(Ae,Ag)). As shown in Figure 1B, cumulus cells from normal-weight patients (BMI: 18.5–23.9 kg/m^2^) significantly enhanced the maturation rate of immature oocytes (0.768 ± 0.019 vs. 0.710 ± 0.022, *p* < 0.05), with a notable improvement observed in GV-stage oocytes (0.689 ± 0.025 vs. 0.599 ± 0.029, *p* < 0.05). Conversely, cumulus cells from overweight/obese patients (BMI ≥ 24 kg/m^2^) significantly reduced the maturation rate of immature oocytes (0.607 ± 0.032 vs. 0.776 ± 0.027, *p* < 0.001), showing a marked decline in GV-stage oocytes (0.546 ± 0.038 vs. 0.728 ± 0.036, *p* < 0.001) (Figure 1C). These findings indicate that cumulus cells from normal-weight patients can significantly promote oocyte maturation, whereas those from overweight or obese patients may impair oocyte developmental competence.

### 2.2. Hyperinsulinemia Exacerbates Overweight/Obesity-Induced Oocyte Maturation Defects

**Figure 2:**
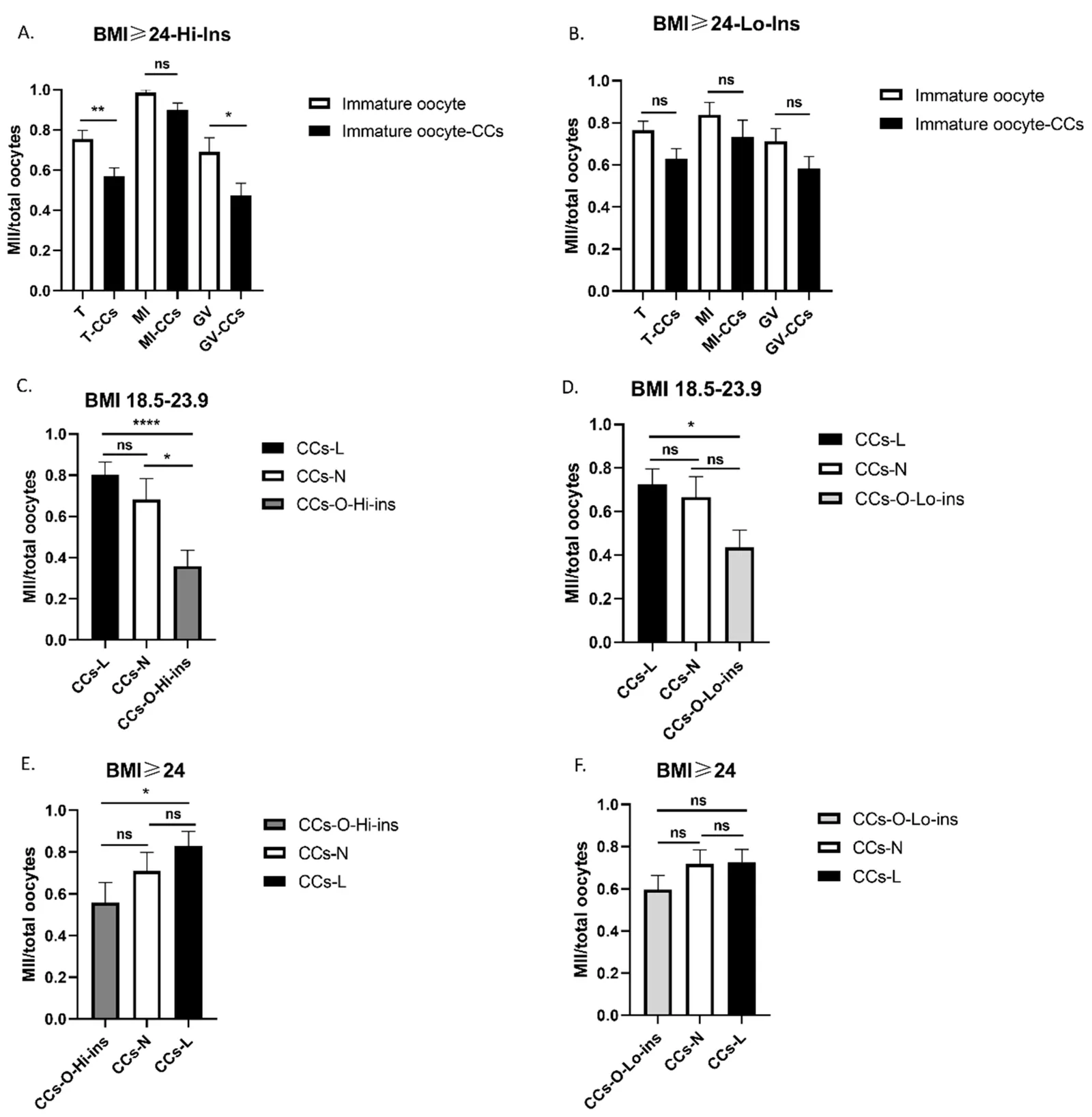
Effects of co-cultured cumulus cells from different BMIs and fasting insulin levels on in vitro oocyte maturation. Patients were stratified into three groups: the normal-weight group (BMI 18.5 kg/m^2^ ≤ BMI < 24.0 kg/m^2^ and fasting insulin ≤ 15 µU/mL), the CCs-O-Hi-Ins group (BMI ≥ 24 kg/m^2^ and fasting insulin > 15 µU/mL), and the CCs-O-Lo-Ins group (BMI ≥ 24 kg/m^2^ and fasting insulin ≤ 15 µU/mL). (**A**) Oocyte maturation following co-culture with autologous cumulus cells from the CCs-O-Hi-Ins group (MI: *n* = 35, *N* = 37; MI-CCs: *n* = 35, *N* = 37; GV: *n* = 41, *N* = 74; GV-CCs: *n* = 59, *N* = 103). (**B**) Oocyte maturation following co-culture with autologous cumulus cells from the CCs-O-Lo-Ins group (MI: *n* = 34, *N* = 46; MI-CCs: *n* = 30, *N* = 43; GV: *n* = 48, *N* = 153; GV-CCs: *n* = 65, *N* = 175). (**C**) Maturation rates of GV-stage oocytes from normal-weight patients following co-culture with cumulus cells from normal-weight patients (CCs-L, *n* = 39, *N* = 48), no cumulus cells added (CCs-N, *n* = 21, *N* = 28), or obese and high-insulin patients (CCs-O-Hi-Ins, *n* = 39, *N* = 45). (**D**) Maturation rates of GV-stage oocytes from normal-weight patients following co-culture with CCs-L (*n* = 38, *N* = 44), CCs-N (*n* = 24, *N* = 28), or cumulus cells from obese and low-insulin patients (CCs-O-Lo-Ins, *n* = 38, *N* = 45). (**E**) Maturation rates of GV-stage oocytes from obese and Hi-Ins patients following co-culture with autologous CCs-O-Hi-Ins (*n* = 26, *N* = 36), CCs-N (*n* = 19, *N* = 24), or CCs-L (*n* = 26, *N* = 38). (**F**) Maturation rates of GV-stage oocytes from obese and Lo-Ins patients following co-culture with autologous CCs-O- Lo-Ins (*n* = 45, *N* = 58), CCs-N (*n* = 42, *N* = 46), or CCs-L (*n* = 45, *N* = 52). Data are presented as mean ± SEM. Note: *n*, number of independent patients; *N*, total number of oocytes; ns, not significant; * *p* < 0.05, ** *p* < 0.01, **** *p* < 0.0001.

To assess the effect of different fasting insulin levels on cumulus cell function in overweight/obese patients, immature oocytes were co-cultured with cumulus cells from patients with a BMI ≥ 24 kg/m^2^, stratified by fasting insulin concentration. The results showed that within the BMI ≥ 24 kg/m^2^ cohort, cumulus cells from patients with low insulin levels (Lo-Ins: fasting insulin level ≤ 15 μU/mL) induced a non-significant reduction in oocyte maturation rates (Figure 2B). However, cumulus cells from patients with high insulin levels (Hi-Ins: fasting insulin level > 15 μU/mL) resulted in a significant decrease in both total and GV-stage oocyte maturation rates (0.569 ± 0.043 vs. 0.754 ± 0.043, *p* < 0.01; 0.475 ± 0.061 vs. 0.691 ± 0.070, *p* < 0.05; Figure 2A). Subsequent rescue experiments were performed to determine whether cumulus cells derived from normal-weight patients (CCs-L) or from BMI ≥ 24 kg/m^2^ patients with low fasting insulin (CCs-O-Lo-Ins) could improve in vitro oocyte maturation.

As shown in Figure 2C, within the normal-weight cohort (BMI 18.5–23.9 kg/m^2^), the proportion of MII oocytes was significantly higher in the CCs-L and CCs-N groups compared to the compromised CCs-O-Hi-Ins group (0.803 ± 0.061 vs. 0.359 ± 0.076, *p* < 0.0001 and 0.682 ± 0.102 vs. 0.359 ± 0.076, *p* < 0.05, respectively). A similar but less pronounced deficit was observed with CCs-O-Lo-Ins, which yielded significantly fewer MII oocytes than the CCs-L group (0.434 ± 0.081 vs. 0.724 ± 0.072, *p* < 0.05; Figure 2D). For GV-stage oocytes from patients with a BMI ≥ 24 kg/m^2^, the maturation rate with CCs-L co-culture was significantly higher than that with CCs-O-Hi-Ins group co-culture (0.827 ± 0.073 vs. 0.558 ± 0.097, *p* < 0.05; Figure 2E), while there was no significant difference in maturation rates between the CCs-L and CCs-O-Lo-Ins groups (Figure 2F). Collectively, these results reveal that obesity combined with hyperinsulinemia exerts the most potent inhibitory effect on oocyte maturation, particularly at the GV stage. This suppression can be effectively reversed by co-culturing with cumulus cells from normal-weight individuals.

### 2.3. Transcriptomic Profiling Reveals Immune Activation and MAPK Pathway Enrichment in Cumulus Cells of Hyperinsulinemic Overweight/Obese Patients

**Figure 3:**
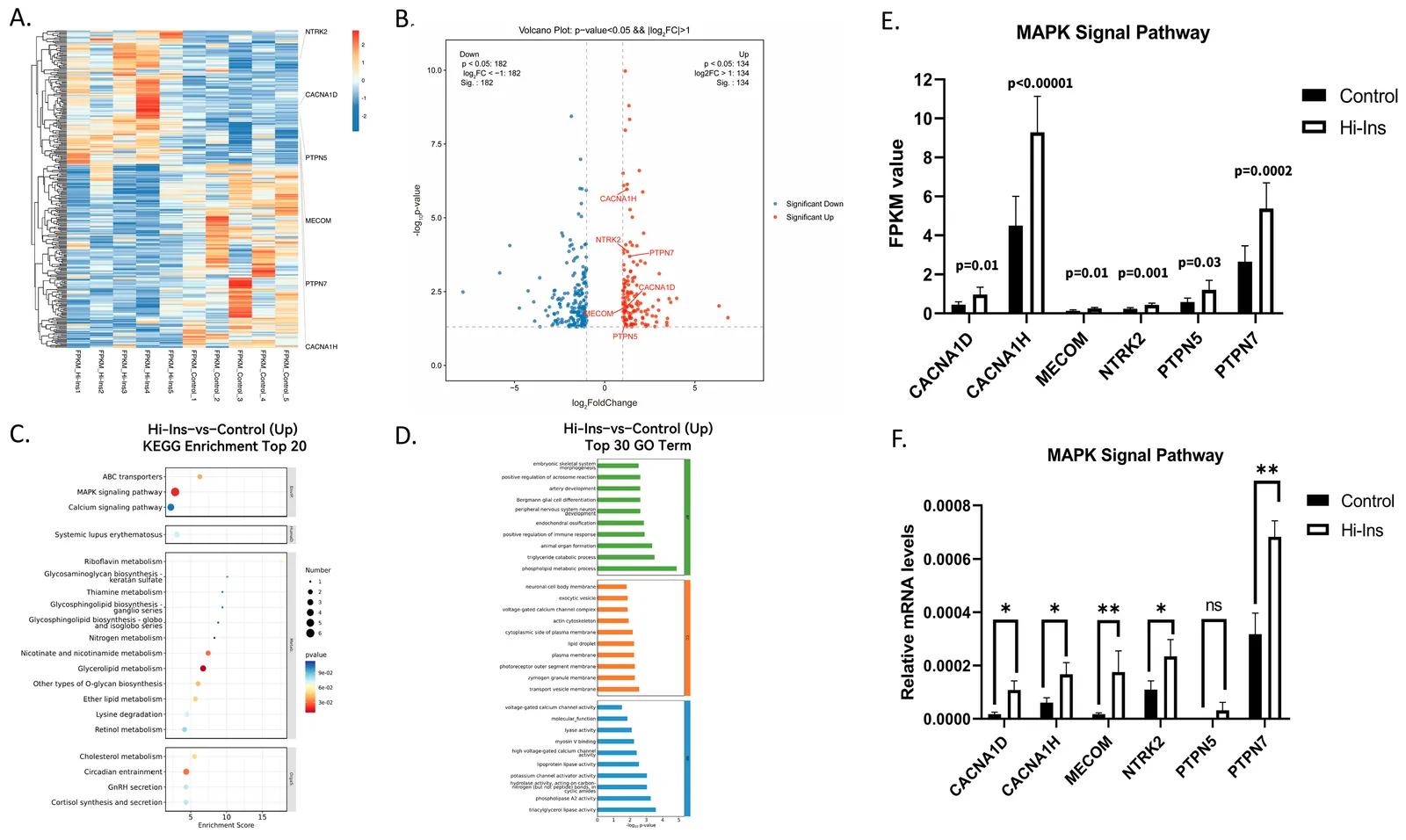
Transcriptomic profiling of cumulus cells (CCs) from normal-weight and obese hyperinsulinemic patients. CCs from obese patients with hyperinsulinemia (BMI ≥ 24 kg/m^2^, fasting insulin > 15 μU/mL, *n* = 5) and normal-weight controls (18.5 kg/m^2^ ≤ BMI < 24.0 kg/m^2^, insulin ≤ 15 μU/mL, *n* = 5) were analyzed. (**A**) Clustering heatmap of differentially expressed genes (DEGs; |log_2_FC| > 1, FDR < 0.05). (**B**) Volcano plot of DEGs; red and blue dots indicate significantly upregulated and downregulated genes, respectively. (**C**) Top 20 up-regulated KEGG pathways, filtered by population hits (PopHits ≥ 5) and ranked by enrichment score. Bubble size indicates gene count; color indicates significance (−log_10_(*p*)). (**D**) Top 30 up-regulated GO terms, ranked by significance. (**E**,**F**) Validation of representative MAPK pathway target genes (*CACNA1D*, *CACNA1H*, *MECOM*, *NTRK2*, *PTPN5*, *PTPN7*) using RNA-seq (**E**) and qPCR (**F**). Data are presented as mean ± SEM. Note: *n*, number of independent patients; ns, not significant; * *p* < 0.05, ** *p* < 0.01.

Transcriptomic profiling identified 316 differentially expressed genes (DEGs) in cumulus cells from patients with BMI ≥ 24 kg/m^2^ and hyperinsulinemia compared to controls (Figure 3A,B, *p* < 0.05, fold change ≥ 2), comprising 134 upregulated and 182 downregulated genes. KEGG analysis revealed significant enrichment of these DEGs in pathways related to immune stress and metabolic dysregulation, with the MAPK signaling pathway being the most prominently enriched (Figure 3C). Gene Ontology (GO) analysis further categorized these DEGs into Biological Process, Cellular Component, and Molecular Function Domains (Figure 3D). Notably, “positive regulation of immune response” was the top enriched term in the Biological Process category. Collectively, these results indicate that the hyperinsulinemic overweight/obese cohort exhibits a state of heightened immune activation and metabolic dysregulation.

To validate these findings, six key candidate genes within the MAPK pathway (*CACNA1D*, *CACNA1H*, *MECOM*, *NTRK2*, *PTPN5*, and *PTPN7*) were selected for qRT-PCR validation. The expression patterns of the other five genes were consistent with the sequencing data. Although *PTPN5* exhibited a consistent trend, the difference did not reach statistical significance (Figure 3E,F).

### 2.4. Altered Inflammatory Profile of Follicular Fluid in Overweight/Obese with Hyperinsulinemia

**Figure 4:**
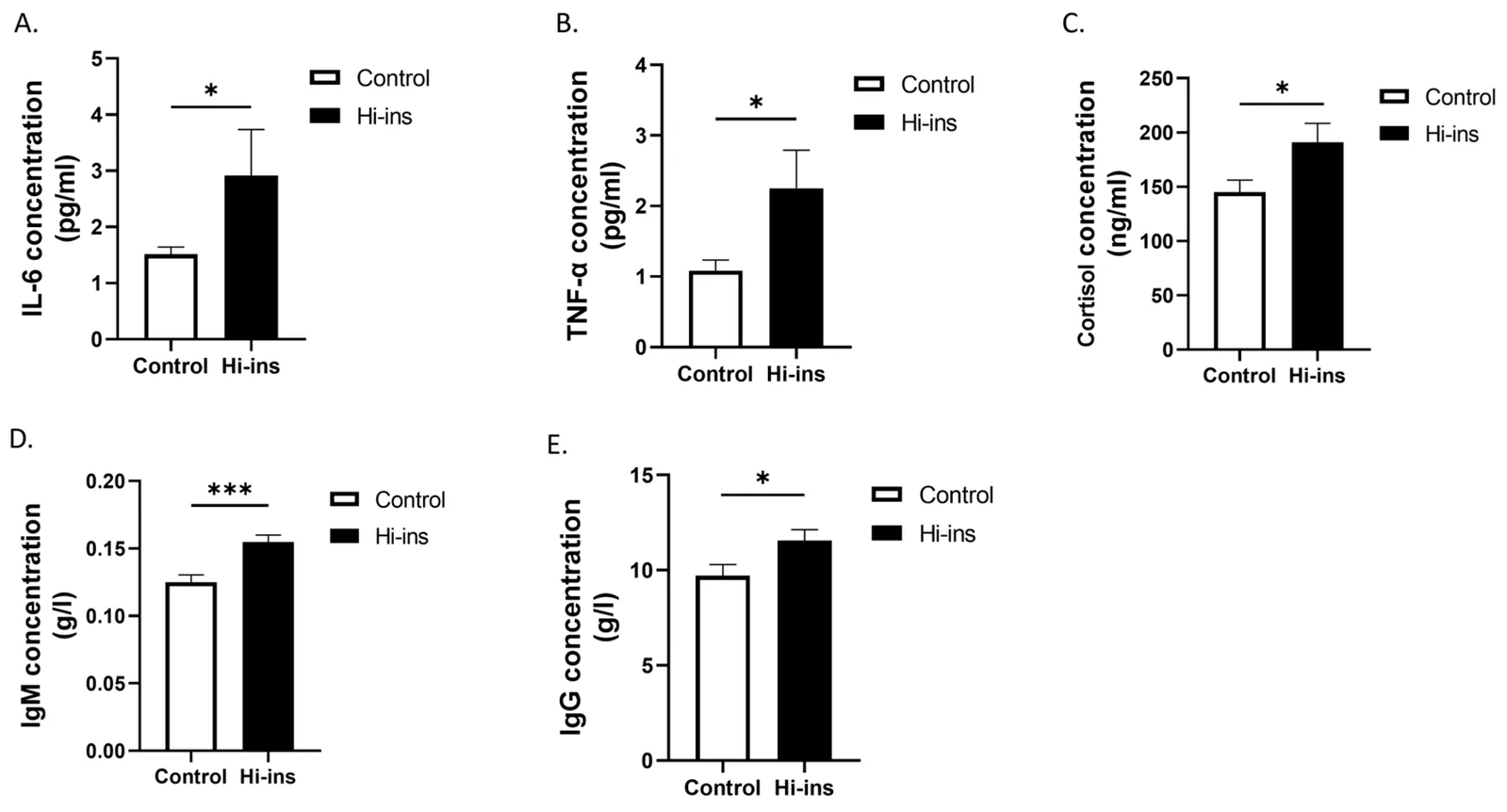
Alteration of inflammation-related markers in follicular fluid (FF) between normal-weight and high-insulin obese patients. Patients were divided into two groups: the control group (18.5 ≤ BMI < 24.0 kg/m^2^, fasting insulin ≤ 15 μU/mL, *n* = 10) and the Hi-Ins group (BMI ≥ 24 kg/m^2^, fasting insulin > 15 μU/mL, *n* = 10). Levels of IL-6 (**A**), TNF-α (**B**), cortisol (**C**), IgM (**D**), and IgG (**E**) were compared between the two groups. Data are presented as mean ± SEM. Note: *n*, number of independent patients; * *p* < 0.05, *** *p* < 0.001.

To determine whether immune stress within the cumulus cells alters follicular fluid composition in overweight/obese patients with hyperinsulinemia, the concentrations of IL-6, TNF-α, cortisol, IgG, and IgM were quantified using ELISA. The results showed that patients with BMI ≥ 24 kg/m^2^ and hyperinsulinemia exhibited significantly elevated levels of the pro-inflammatory cytokines IL-6 and TNF-α compared to normal-weight controls (IL-6: 2.92 ± 0.82 pg/mL vs. 1.51 ± 0.13 pg/mL, *p* < 0.05; TNF-α: 2.25 ± 0.54 pg/mL vs. 1.09 ± 0.15 pg/mL, *p* < 0.05, Figure 4A,B). This systemic inflammatory state was further evidenced by markedly higher cortisol levels in the Hi-Ins group than in the control group (191.00 ± 17.37 ng/mL vs. 145.10 ± 11.11 ng/mL, *p* < 0.05, Figure 4C). Moreover, compared to the control group, the level of IgG was significantly elevated in the Hi-Ins cohort (11.55 ± 0.58 g/L vs. 9.72 ± 0.57 g/L, *p* < 0.05, Figure 4E). Notably, the IgM concentrations in follicular fluid significantly increased in the Hi-Ins group compared to controls (Hi-Ins group: 0.155 ± 0.005 g/L vs. 0.125 ± 0.005 g/L, *p* < 0.001, Figure 4D).

Collectively, these findings demonstrate that the follicular inflammatory profile is remodeled in overweight/obese patients with hyperinsulinemia, characterized by elevated levels of pro-inflammatory cytokines, cortisol, and immunoglobulins.

### 2.5. The Antioxidant N-Acetyl-L-Cysteine Improves Oocyte Maturation in Overweight/Obese Patients

**Figure 5:**
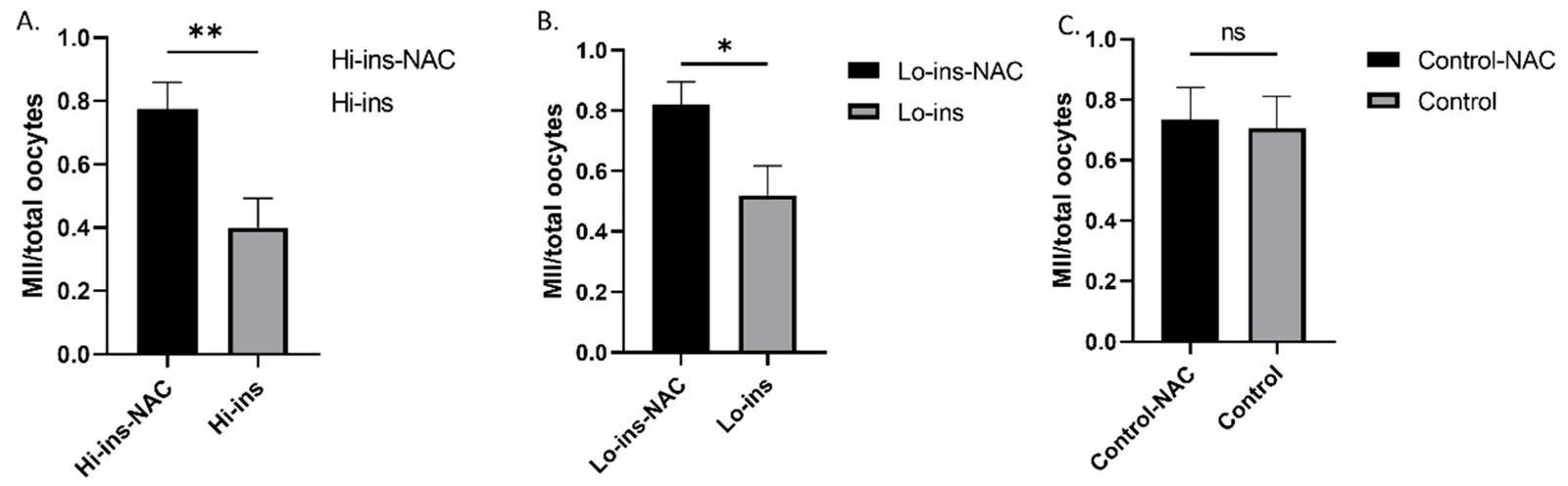
The effect of N-acetyl-L-cysteine (NAC) on oocyte maturation during in vitro co-culture. Immature oocytes at the germinal vesicle (GV) stage were co-cultured with cumulus cells at 37 °C, 5% O_2_, 6% CO_2_, and balanced with N_2_ for 24 h, with or without 0.3 mM NAC. (**A**) Co-culture of GV-stage oocytes with autologous cumulus cells from the Hi-Ins group, supplemented with 0.3 mM NAC (*n* = 20, *N* = 32). (**B**) Co-culture of GV-stage oocytes with autologous cumulus cells from the Lo-Ins group, supplemented with 0.3 mM NAC (*n* = 25, *N* = 32). (**C**) Co-culture of GV-stage oocytes with autologous cumulus cells from the control group, supplemented with 0.3 mM NAC (*n* = 17, *N* = 26). Data are presented as mean ± SEM. Note: *n*, number of independent patients; *N*, total number of oocytes; ns, not significant; * *p* < 0.05, ** *p* < 0.01.

To evaluate the therapeutic potential of antioxidant intervention, GV-stage oocytes were co-cultured with autologous cumulus cells and treated with 0.3 mM N-acetyl-L-cysteine (NAC) at 37 °C, 5% O_2_, 6% CO_2_, and balanced with N_2_ for 24 h. The results revealed that NAC supplementation significantly enhanced the in vitro maturation of GV-stage oocytes when co-cultured with autologous cumulus cells from overweight/obese individuals. This restorative effect on oocyte maturation was most evident in the Hi-Ins subgroup (0.775 ± 0.085 vs. 0.400 ± 0.093, *p* < 0.01, Figure 5A). Furthermore, a notable improvement in oocyte maturation was also observed in the Lo-Ins subgroup (0.820 ± 0.076 vs. 0.520 ± 0.098, *p* < 0.05, Figure 5B). Notably, this rescue effect did not occur in the control GV-stage oocyte group, which was co-cultured with autologous cumulus cells from normal-weight individuals (0.735 ± 0.106 vs. 0.706 ± 0.106, *p* > 0.05, Figure 5C), indicating that NAC could specifically counteract the pathological follicular environment induced by overweight/obesity.

## 3. Discussion

As somatic cells in direct contact with the oocyte, cumulus cells provide metabolic and signaling cues essential for oocyte development. Evidence suggests that co-culturing oocytes with autologous cumulus cells during IVM partially reconstitutes the follicular microenvironment, leading to improved nuclear maturation and, crucially, the restoration of a transcriptional profile more closely resembling that of in vivo-matured oocytes [22]. However, the specific mechanisms by which cumulus cells from patients with overweight/obesity and hyperinsulinemia compromise oocyte maturation remain largely unexplored.

Our findings demonstrate that supplementing the IVM system with cumulus cells from normal-weight women significantly enhances the maturation rate of immature oocytes. In contrast, co-culture with cumulus cells derived from overweight/obese patients (BMI ≥ 24 kg/m^2^, based on Chinese criteria) compromises oocyte maturation. These in vitro results highlight a distinct regulatory capacity of cumulus cells based on donor BMI, with cumulus cells from high-BMI women exerting a particularly detrimental influence.

Hyperinsulinemia is a core feature of obesity and associated metabolic dysregulation. Although fasting serum and follicular fluid insulin levels correlate positively with BMI in obese women [13], the impact of hyperinsulinemia on cumulus cell function in vitro remains poorly characterized. Herein, our study identified hyperinsulinemia (fasting insulin > 15 µU/mL) as a critical driving factor for cumulus cell dysfunction in obese patients. Specifically, in the BMI ≥ 24 kg/m^2^ subgroup, cumulus cells derived from hyperinsulinemic donors significantly compromised the maturation rate of GV-stage oocytes (from 72.8% to 54.6%), whereas those from normoinsulinemic donors exerted no significant inhibitory effect. These findings confirm that hyperinsulinemia serves as a key amplifier of cumulus cell dysfunction, thereby impairing oocyte maturation.

Prior studies in human granulosa tumor cells (KGN) showed that combined exposure to high free fatty acids (FFAs) and insulin was more deleterious than FFA treatment alone. This co-treatment significantly increased apoptotic signaling and upregulated the expression of pro-inflammatory cytokines and oxidative stress markers, characterized by the concurrent activation of multiple canonical pathways involving IL-6, NF-κB, and RANK signaling [23]. Moreover, our human transcriptomic data corroborate these mechanisms, demonstrating that cumulus cells from hyperinsulinemic patients with a BMI ≥ 24 kg/m^2^ display significant enrichment of the MAPK signaling pathway and upregulated immune response pathways. Recent evidence indicates that pro-inflammatory signaling and oxidative stress markers are closely intertwined [24]. In this context, hyperinsulinemia can induce excessive reactive oxygen species (ROS) production, which in turn stimulates cumulus cells to release more IL-6 and TNF-α [25]. This pro-inflammatory state was clinically evidenced by positive correlations between circulating IL-6 and insulin resistance [26]. Consequently, this interaction establishes a self-perpetuating vicious cycle that ultimately compromises normal follicular development.

The mitogen-activated protein kinase (MAPK) pathway is a highly conserved signaling cascade in eukaryotes that transduces extracellular stimuli into intracellular responses. Upon inflammatory stimulation, MAPK activation regulates the production of pro-inflammatory cytokines (e.g., TNF-α, IL-6, IL-1β), chemokines, and adhesion molecules, as well as modulating immune cell function and differentiation [27]. This transcriptional signature aligns with recent evidence highlighting the pivotal role of the MAPK pathway in regulating these cytokines. For instance, the accumulation of free cholesterol in macrophages during advanced atherosclerosis activates MAPK, thereby inducing TNF-α and IL-6 secretion [28]. Furthermore, studies in lung epithelial models confirm that p38 inhibition eliminates TNF-α and IL-6 release, and NAC-mediated suppression of p38 phosphorylation provides a direct antioxidant-based strategy to curb this inflammatory output [29]. Within the follicle, this transcriptomic and biochemical shift translates into functional impairment. Given that CACNA1D couples Ca^2+^ signaling to MAPK-mediated inflammation in myeloid cells [30], its dysregulation in cumulus cells may similarly amplify oxidative stress and compromise oocyte maturation. Furthermore, pathologically elevated IL-6 acts as a key autocrine disruptor in cumulus cells, directly damaging oocytes by disrupting spindle microtubule organization and chromosomal alignment [31,32,33]. Similarly, TNF-α, primarily secreted by cumulus cells, macrophages, and oocytes, exerts concentration-dependent toxicity. At high concentrations, it engages both TNFR1 and TNFR2 to trigger granulosa cell apoptosis [34]. This molecular signature strongly suggests that cumulus cells from overweight/obese patients with hyperinsulinemia exhibit dysregulated immune activation, thereby disrupting the follicular microenvironment and compromising oocyte maturation.

Our RNA-seq analysis revealed significant enrichment of MAPK signaling components in cumulus cells from the Hi-Ins group, providing a molecular basis for the elevated IL-6 and TNF-α levels detected in the corresponding follicular fluid. Existing evidence confirms that granulosa cell dysfunction drives the pathological shifts observed in the follicular fluid microenvironment [17]. Consistent with this, our findings demonstrate that the follicular fluid of obese patients with hyperinsulinemia contains significantly elevated levels of IL-6 and TNF-α compared with normal-weight controls. Intriguingly, cortisol, a steroid hormone with potent immunosuppressive and anti-inflammatory properties [34], was also significantly increased, suggesting a state of follicular inflammatory stress accompanied by a compensatory activation of the hypothalamic–pituitary–adrenal (HPA) axis. Collectively, these findings demonstrate that the follicular inflammatory profile is remodeled in overweight/obese patients with hyperinsulinemia, characterized by elevated levels of pro-inflammatory cytokines and cortisol.

Accumulating evidence indicates that dysfunctional cumulus cells impair oocyte quality by inducing endoplasmic reticulum stress, oxidative stress, and mitochondrial dysfunction. These disruptions collectively compromise meiotic maturation and spindle morphology, ultimately diminishing developmental competence [35,36]. Supporting this notion, our functional assays demonstrate that cumulus cells from overweight/obese patients, particularly those with hyperinsulinemia, directly compromise the maturation of GV oocytes derived from normal-weight patients. Importantly, this impairment was reversible; co-culture with cumulus cells from normal-weight patients significantly rescued the maturation of GV oocytes from obese women (BMI ≥ 24 kg/m^2^).

Obesity is characterized by chronic, low-grade systemic inflammation [37], in which IgG acts as a key pathogenic driver. FcRn-mediated IgG accumulation promotes macrophage infiltration and pro-inflammatory cytokine secretion [38]. Furthermore, IgG functions as a natural inhibitor of insulin signaling; its Fc-CH3 domain competitively binds to the insulin receptor, directly inducing insulin resistance and subsequent compensatory hyperinsulinemia [39]. In alignment with this systemic profile, we observed a selective elevation of follicular IgG specifically in hyperinsulinemic overweight/obese patients, concurrent with an increase in pro-inflammatory cytokines.

Conversely, IgM serves as a crucial counter-regulatory mechanism. Saturated fatty acids can stimulate the production of serum IgM via TLR4 activation [40], which recognizes oxidation-specific epitopes arising from inflammation and cell death. Subsequent binding inhibits TNF-α and MCP-1 production by M1 macrophages, thereby attenuating visceral adipose tissue inflammation and improving insulin sensitivity [41]. In this study, follicular fluid IgM was markedly elevated in overweight/obese patients with hyperinsulinemia (Hi-Ins). We propose that this upregulation represents a compensatory response to scavenge oxidation-specific epitopes and counteract the heightened inflammatory milieu. However, the oxidative and inflammatory burden in the Hi-Ins cohort—evidenced by significantly elevated cortisol and pro-inflammatory cytokines—was substantially greater than in normal-weight controls. Consequently, despite this IgM-mediated compensatory attempt, the underlying pathological load appeared to overwhelm the system, resulting in decompensated follicular inflammation.

N-acetyl-L-cysteine (NAC), a potent antioxidant, enhances total antioxidant capacity by replenishing intracellular glutathione levels [42] and directly scavenging free radicals, such as hydrogen peroxide and superoxide [43]. Moreover, it exerts significant anti-inflammatory effects by inhibiting the release of pro-inflammatory cytokines, including TNF-α and various interleukins [44]. Clinically, NAC is a well-tolerated mucolytic agent that has been shown to improve insulin sensitivity [45]. Crucially, NAC rescues oocyte maturation impaired by environmental stressors such as heat shock, bisphenol A, and zearalenone, while protecting spindle architecture [46,47,48,49]. Furthermore, our functional assays demonstrate that supplementing the IVM medium with a cytocompatible and effective concentration of NAC (0.3 mM) significantly rescued the maturation of GV oocytes co-cultured with autologous cumulus cells from either normoinsulinemic or hyperinsulinemic obese patients. These findings indicate that NAC mitigates the in vitro toxicity driven by obesity-associated cumulus cell inflammation and oxidative stress, thereby overcoming the impairment in oocyte maturation.

**Figure 6:**
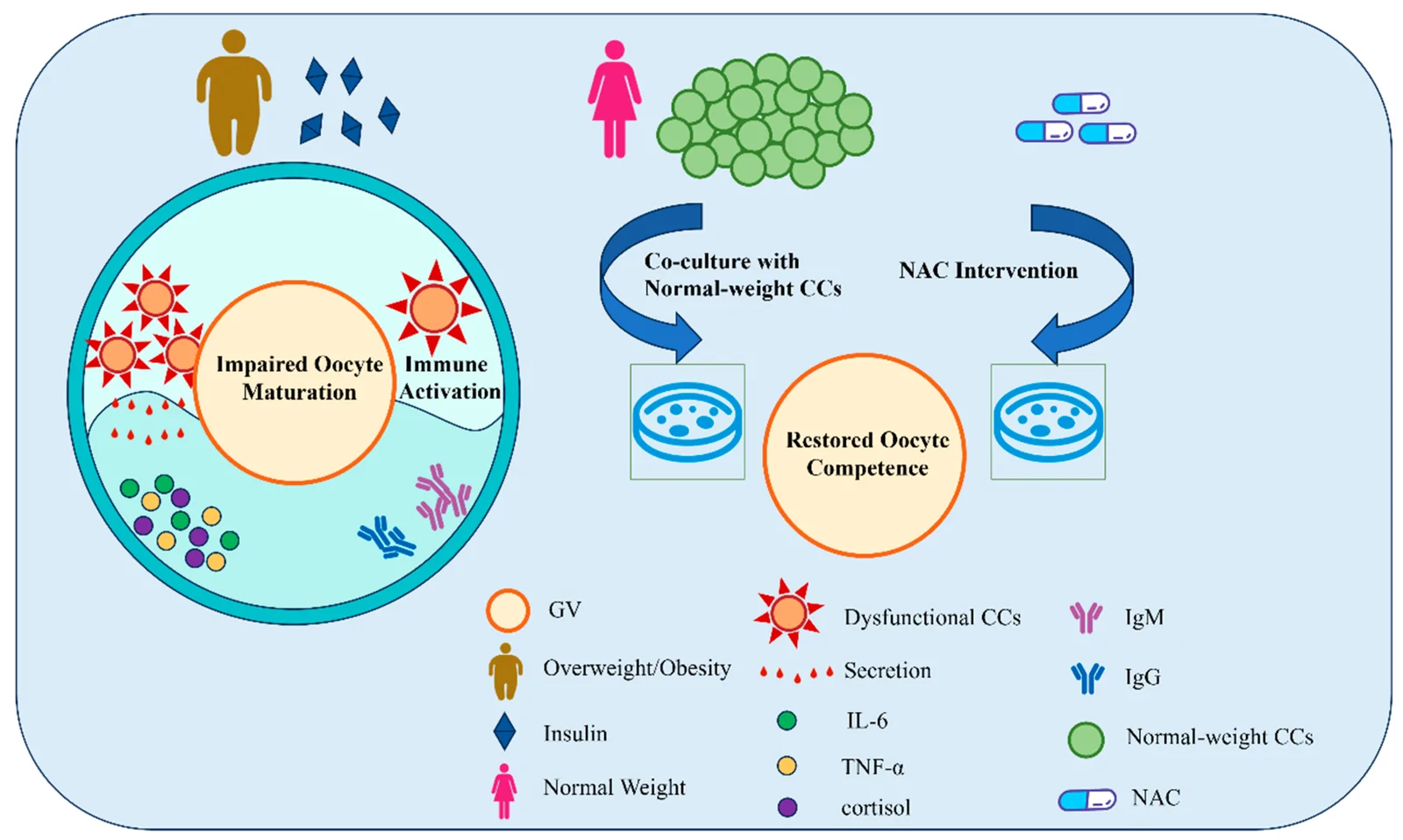
Schematic model illustrating the mechanism by which hyperinsulinemia drives obesity-associated cumulus cell dysfunction and oocyte developmental failure, and the rescue of oocyte competence by in vitro co-culture interventions. In hyperinsulinemic obese females, the cumulus–oocyte complex undergoes pathological remodeling, characterized by elevated pro-inflammatory cytokines (IL-6, TNF-α), immunoglobulins (IgG, IgM), and cortisol. This pro-oxidative and pro-inflammatory milieu impairs cumulus cell function. Consequently, co-culture with these dysfunctional cumulus cells significantly compromises the in vitro maturation of immature oocytes, particularly at the GV stage. Notably, this impairment is effectively reversed by co-culture with healthy cumulus cells from normal-weight patients or by NAC supplementation, underscoring the therapeutic potential of optimizing the peri-oocyte environment.

In summary, our findings indicate that cumulus cell dysfunction stems from the synergistic interplay between obesity and hyperinsulinemia. Although hyperinsulinemia likely exacerbates underlying metabolic disturbances, the combined obese and hyperinsulinemic phenotype acts as the primary disruptor of the follicular microenvironment, driving oxidative stress and impairing in vitro oocyte maturation. Notably, these defects were partially reversible via co-culture with healthy cumulus cells or NAC supplementation (Figure 6). Given the scarcity of human samples, our analysis was restricted to nuclear maturation and transcriptomics, precluding the evaluation of cytoplasmic markers or gap junction functionality. Therefore, our conclusions specifically address the impact of the obesity–hyperinsulinemia phenotype on nuclear competence and cumulus cell gene expression. Future studies utilizing animal models are warranted to comprehensively elucidate the associated cytoplasmic and metabolic deficits.

## 4. Materials and Methods

### 4.1. Patient Selection and Specimen Collection

All human study procedures were approved by the Medical Research Ethics Committee of Ren Ji Hospital, Shanghai Jiao Tong University School of Medicine (KY2024-189-B; Approval Date: 16-December-2024), and written informed consent was obtained from all participants. This study complied with the Declaration of Helsinki. Based on Chinese population characteristics, obesity diagnosis followed the national criteria: Normal weight: 18.5 ≤ BMI < 24.0 kg/m^2^; Overweight: 24.0 ≤ BMI < 28.0 kg/m^2^; and Obesity: BMI ≥ 28.0 kg/m^2^ [50]. Detailed baseline characteristics of the enrolled patients are summarized in Supplementary Material S1 (Table S1).

Furthermore, according to the 2024 Chinese Diabetes Prevention and Treatment Guidelines [51], hyperinsulinemia was defined as a fasting insulin level > 15 μU/mL, with levels ≤ 15 μU/mL considered normal. This threshold is widely applied as a screening indicator for insulin resistance in the Chinese population.

Follicular fluid (FF) and cumulus cells were obtained from patients undergoing in vitro fertilization (IVF). All participants underwent controlled ovarian stimulation using a gonadotropin-releasing hormone (GnRH) antagonist protocol. Once the leading follicles reached an appropriate size based on predefined criteria, final oocyte maturation was triggered by administering human chorionic gonadotropin (hCG; Livzon Pharmaceutical Group, Zhuhai, China). Transvaginal ultrasound-guided oocyte retrieval was performed 36 h post-trigger. FF was aspirated from follicles of uniform size (18–20 mm in diameter) and pooled for subsequent processing. Cumulus cells were harvested from cumulus-oocyte complexes (COCs) via gentle mechanical scraping with a Pasteur pipette under a stereomicroscope during oocyte retrieval. Immediately after collection, the cells were transferred to IVF culture medium (Vitrolife, Gothenburg, Sweden). They were used directly for co-culture without prior culture, passaging, or enzymatic digestion. The experimental groups were defined as follows:

A. Hi-Ins group: BMI ≥ 24 kg/m^2^ and fasting insulin level > 15 μU/mL;
B. Lo-Ins group: BMI ≥ 24 kg/m^2^ and fasting insulin level ≤ 15 μU/mL;
C. Normal-weight/Control group: 18.5 ≤ BMI < 24 kg/m^2^ with fasting insulin level ≤ 15 μU/mL.

### 4.2. In Vitro Maturation

Cumulus-oocyte complexes (COCs) were denuded 2–5 h post-retrieval using 80 IU/mL hyaluronidase (Hyaluronidase™, Origio, Måløv, Denmark) combined with gentle mechanical pipetting. Oocyte maturity was assessed morphologically: oocytes exhibiting the extrusion of a first polar body were classified as the MII stage. Oocytes with an intact germinal vesicle (GV) were classified as immature (GV stage), while those lacking both an intact germinal vesicle and a first polar body were classified as the MI stage.

In vitro maturation (IVM) was initiated exclusively for patients from whom ≥ 2 immature oocytes (MI or GV) were retrieved. The immature oocytes were randomly and equally allocated into each experimental (*n* = 1–4) and control (*n* = 1–4) group in 4-well plates (0.7 mL per well). The oocytes were cultured in G_1_ medium (90.08 mM NaCl, 5.5 mM KCl, 0.25 mM NaH_2_PO_4_, 1.0 mM MgSO_4_, 25.0 mM NaHCO_3_, 1.8 mM CaCl_2_, 0.5 mM glucose, 10.5 mM sodium lactate, 0.32 mM pyruvate, supplemented with amino acids and other essential nutrients) at 37 °C in an atmosphere of 5% O_2_, 6% CO_2_, and balanced N_2_ either with or without cumulus cells (0.5–0.8 × 10^5^ cells), for 24 h.

### 4.3. Gene Expression Microarray Profiles of Cumulus Cells

Ten patients were enrolled in this study, consisting of five overweight/obese individuals (BMI 24–34 kg/m^2^; fasting insulin > 15 μU/mL) and five normal-weight controls (BMI 18.5–23.9 kg/m^2^; fasting insulin ≤ 15 μU/mL).

Cumulus cells were collected during oocyte retrieval by mechanically stripping cumulus-oocyte complexes under a stereomicroscope. The collected cells were then isolated via density gradient centrifugation using 1.077 g/mL Ficoll (Cytiva, Wilmington, DE, USA) at 1500× *g* for 25 min. The cell pellets were washed three times with PBS (Gibco, NY, USA) and lysed in TRIzol™ reagent (Invitrogen, Carlsbad, CA, USA), followed by storage at −80 °C until RNA extraction.

Total RNA was extracted using TRIzol reagent (Invitrogen, Carlsbad, CA, USA) following the manufacturer’s protocol. RNA purity and concentration were measured using a NanoDrop 2000 spectrophotometer (Thermo Fisher Scientific, Waltham, MA, USA), and RNA integrity was assessed using an Agilent 2100 Bioanalyzer (Agilent Technologies, Santa Clara, CA, USA). (Supplementary Material S2, Supplementary Figure S1). RNA concentration was precisely quantified, and an input of 0.5 µg total RNA was uniformly used for library preparation across all samples to ensure experimental consistency. Sequencing libraries were prepared with the VAHTS Universal V10 RNA-seq Library Prep Kit (Premixed Version) in accordance with the manufacturer’s instructions. Transcriptome sequencing and subsequent bioinformatic analysis were performed by OE Biotech Co., Ltd. (Shanghai, China) on an Illumina NovaSeq 6000 platform.

Sequencing was performed on an Illumina NovaSeq 6000 platform to generate 150 bp paired-end reads. Raw sequencing data in FASTQ format were first processed with fastp (v0.20.1) for quality control to remove low-quality reads and generate clean reads. Clean reads were then aligned to the reference genome GRCh38.p13 using HISAT2 (v2.1.0). Raw read counts per gene were derived using HTSeq-count (v0.11.2), and gene expression levels were subsequently quantified as FPKM. Principal Component Analysis (PCA) was conducted in R (v3.2.0) to assess biological reproducibility across samples. Differential expression analysis was performed with DESeq2 (v1.22.2). Genes meeting the criteria of an adjusted *p*-value < 0.05 and absolute log2 fold change > 1 were classified as significantly differentially expressed genes (DEGs). The raw sequence data have been deposited in the Genome Sequence Archive (GSA-Human) at the National Genomics Data Center, China National Center for Bioinformation/Beijing Institute of Genomics, Chinese Academy of Sciences (GSA-Human: HRA019282; BioProject: PRJCA066664).

### 4.4. Quantitative RT-PCR (qRT-PCR)

Total RNA was extracted from cumulus cells using TRIzol™ reagent (Invitrogen, Carlsbad, CA, USA) according to the manufacturer’s protocol. For each sample, 0.5 µg of total RNA was reverse-transcribed into first-strand cDNA using the PrimeScript™ RT reagent Kit (Takara Bio, Shiga, Japan). Gene-specific primers (listed in Table 1) were designed using Primer Express Software 3.0.1 (Applied Biosystems, Foster City, CA, USA) based on published cDNA sequences, and their specificity was confirmed via NCBI BLAST (2.17.0) searches. Amplification efficiencies for all primer pairs were validated using standard curves generated from five-fold serial dilutions of pooled cDNA and ranged between 90% and 110% (R^2^ > 0.99; details are provided in Supplementary Material S3).

Quantitative real-time PCR was performed using an ABI 7900HT Sequence Detection System with SYBR™ Green Master Mix (Thermo Fisher Scientific, Waltham, MA, USA) to quantify the mRNA levels of *CACNA1D*, *CACNA1H*, *MECOM*, *NTRK2*, *PTPN5*, *PTPN7*, and the endogenous control *GAPDH*. The thermocycling protocol consisted of an initial hold at 50 °C for 2 min and 95 °C for 10 min, followed by 40 cycles of 95 °C for 15 s and 60 °C for 1 min. Although systematic no-template controls (NTCs) and no-reverse-transcription controls (NRTs) were included in the initial runs, melting curve analysis revealed single-peak amplification for all reactions, confirming assay specificity. All reactions were performed in triplicate. Relative gene expression was normalized to *GAPDH* using the 2^(−ΔΔCt)^ method. The stability of *GAPDH* as a reference gene was further validated using our RT-qPCR data, which showed no significant expression differences between hyperinsulinemic and control groups (Supplementary Material S3).

### 4.5. Human Follicular Fluid Analyses by ELISA

Concentrations of IL-6, TNF-α, cortisol, IgM, and IgG in follicular fluid were quantified using commercial ELISA kits (Elabscience, Wuhan, China) according to the manufacturer’s instructions. Briefly, samples were diluted as follows prior to assay: IL-6 (1:2), TNF-α (undiluted), cortisol (1:2), IgM (1:2000), and IgG (1:400,000). For cortisol detection, 50 µL of diluted sample was added per well; for all other analytes, 100 µL of sample was used. All standards and samples were assayed in duplicate. After incubation and washing steps, the reaction was developed with TMB substrate and stopped with sulfuric acid. Absorbance was measured at 450 nm using a Multiskan MK3 microplate reader (Thermo Fisher Scientific, Waltham, MA, USA). Concentrations were calculated by interpolating sample absorbance values against a standard curve fitted with a four-parameter logistic (4-PL) nonlinear regression model.

### 4.6. Antioxidant Supplementation in IVM Co-Culture Systems

Immature oocytes at the GV stage were co-cultured with autologous cumulus cells in G_1_ medium under standard conditions (37 °C, 5% O_2_, 6% CO_2,_ and balanced with N_2_) for 24 h. To evaluate the effect of antioxidant treatment, N-acetyl-L-cysteine (NAC; MedChemExpress, Monmouth Junction, NJ, USA) was added to the co-culture system at a final concentration of 0.3 mM. Control cultures were maintained under identical conditions without NAC supplementation.

### 4.7. Statistical Analysis

The patient was considered the experimental unit. Oocytes from each patient were randomized for paired comparisons. Maturation rates (GV, MI, and TI) were analyzed using a binomial generalized linear mixed model (GLMM) with patient as a random effect. MI and GV were tested separately with Bonferroni correction (*p* < 0.025). Continuous data were assessed for normality and variance homogeneity prior to analysis. Depending on the data distribution, two-group comparisons were performed using the independent samples *t*-test or Mann–Whitney *U* test, while comparisons among ≥3 groups were analyzed using ANOVA or the Kruskal–Wallis test. Paired and multi-group comparisons were further evaluated using the Wilcoxon signed-rank test or Dunn’s test with appropriate post hoc corrections. Statistical analyses were performed using GraphPad Prism (version 8.0.2, San Diego, CA, USA). Statistical significance was set at *p* < 0.05.

## 5. Conclusions

This study identifies hyperinsulinemia as the pivotal driver of obesity-associated cumulus cell dysfunction and oocyte developmental failure. The coexistence of hyperinsulinemia and obesity synergistically triggers a pathological cascade characterized by elevated inflammatory cytokines (IL-6, TNF-α), IgG, and HPA axis activation. This leads to chronic oxidative and inflammatory stress, impairing cumulus cell function and arresting oocyte maturation. While the observed upregulation of IgM implies a compensatory response against oxidative stress, it fails to fully counteract the damage. Notably, we demonstrate that supplementing the IVM system with healthy cumulus cells or NAC significantly restores oocyte maturation, highlighting its therapeutic potential for hyperinsulinemic obese patients. Collectively, these findings underscore that hyperinsulinemia exacerbates obesity-induced follicular stress, and that targeted microenvironmental interventions can effectively rescue oocyte developmental competence.

## Figures and Tables

**Table 1:** PCR primer sequences.

Items	Primer Sequence (5′-3′)
CACNA1D-F	GAAGCGAGAATAAGGGCAGG
CACNA1D-R	CTCTTGCATAGTTTGCCTCGTT
CACNA1H-F	TAACCAACCCAAGTCGCTGG
CACNA1H-R	ATGCCCGTAGCCATCTTCAG
MECOM-F	ACCCTTTGGCTAGATTGCTTATTC
MECOM-R	TTGAGCCAGCTTCCAACATCT
NTRK2-F	GCAATCCATTTACATGCTCCTGT
NTRK2-R	CATATTAGGAACCGGATCACCTG
PTPN5-F	ATGAGGGAGCCAGGAGTGAG
PTPN5-R	CACGGACAGAAAGACCAGCA
PTPN7-F	CCCAAGCAACTGGAAGAAGAAT
PTPN7-R	TTCCCGTCATAGCCTCGGAT
GAPDH-F	GTCAAGGCAGAGAACGGGAA
GAPDH-R	GGTTCACGCCCATCACAAAC

## Data Availability

The RNA-seq data generated in this study are available from the corresponding author upon reasonable request.

## Supplementary Materials

The following supporting information can be downloaded at https://www.mdpi.com/article/10.3390/ijms27188246/s1.
