# Supplementary material for: Overweight/Obesity and Hyperinsulinemia Impair Oocyte Developmental Competence via Cumulus Cell Dysfunction and Oxidative Stress-Mediated Follicular Microenvironment Remodeling
Source: Int J Mol Sci. 2026 Sep 16;27(18):8246. doi: 10.3390/ijms27188246 (PMC13607404; doi:10.3390/ijms27188246)
Supplement: Supplementary file 1 [file ijms-27-08246-s001.zip › Supplementary Material S2.pdf]

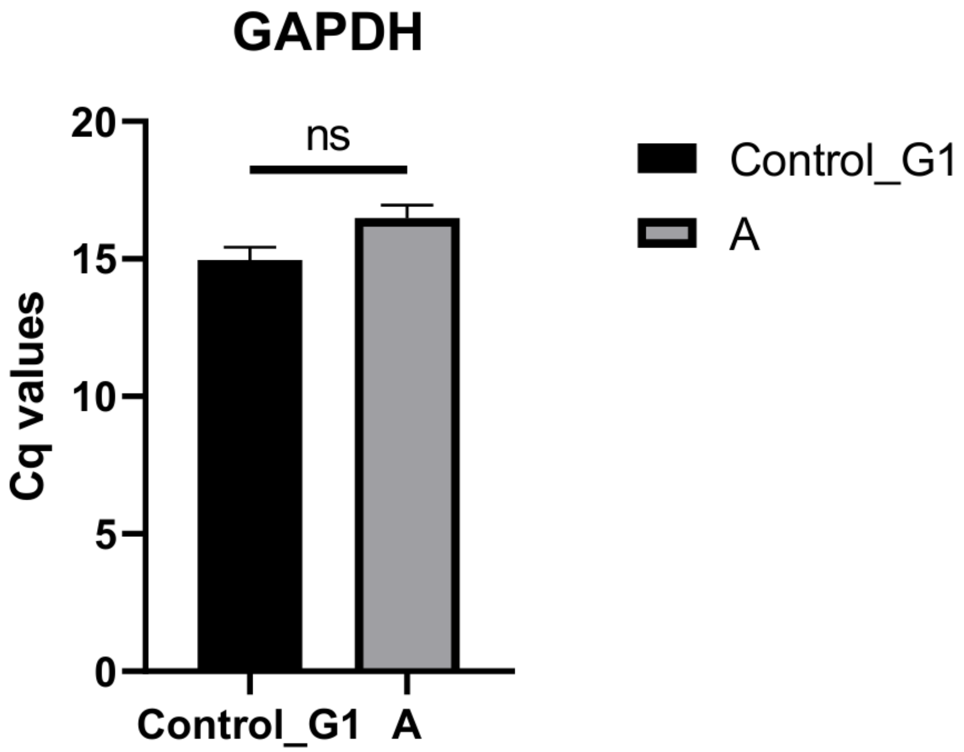

Supplementary Figure S1. GAPDH Cq values in Control_G1 and A groups. Bar graphs show mean ± SEM. GAPDH Cq did not differ significantly between Control_G1(14.96±0.46) and A group (16.48±0.49) (ns, Mann–Whitney U). The low CV (Control_G1:10.7%, A: 12.50%)) and minimal between-group Cq shift support the use of GAPDH as a single reference gene.

Real-Time Fluorescence Quantitative PCR Service Report

Table of Contents

1. Abstract ………………………………………………………….…. 3
2. Materials, Reagents and Instruments ………………………………… 3
3. Experimental Procedures …………………………………………….3
4. Experimental Results ……………………………………………..…. 4
5. Appendix …………………………………………………..… ……. 12
6. Remarks …………………………………………… ………...…. 12

1. Abstract

Real-time fluorescence quantitative PCR (qPCR) employs the fluorescent dye SYBR Green I to monitor PCR amplification in real time via fluorescence accumulation, allowing quantification of the initial template. Relative quantification uses the $2^{(-\Delta\Delta Ct)}$ method, in which the Ct value of the target gene is normalized to that of the reference gene to determine the fold change in expression between samples.

---

2. Materials, Reagents and Instruments

2.1 Main Materials and Reagents

Reagent Name	Supplier	Catalog No.	Remarks
SGExcel FastSYBR Mixture	Sangon Biotech	B532955	

2.2 Main Instruments

Instrument	Manufacturer	Model	Remarks
Clean bench	Jiangsu Sujie Purification Equipment Co.	SW-CJ-1D	
Pipettes (100–1000 µL, 20–200 µL, 0.5–10 µL)	BBI, Canada	—	

Instrument	Manufacturer	Model	Remarks
LightCycler 480 Real-Time PCR System	Roche, Rotkreuz, witzerland	LightCycler 480 II	

3. Experimental Procedures

3.1 Primer Sequences

- GAPDH-F GTCAAGGCAGAGAACGGGAA
- GAPDH-R GGTTCACGCCCATCACAAAC
- CACNA1D-F GAAGCGAGAATAAGGGCAGG
- CACNA1D-R CTCTTGCATAGTTTGCCTCGTT
- CACNA1H-F TAACCAACCCAAGTCGCTGG
- CACNA1H-R ATGCCCGTAGCCATCTTCAG
- MECOM-F ACCCTTTGGCTAGATTGCTTATTC
- MECOM-R TTGAGCCAGCTTCCAACATCT
- NTRK2-F GCAATCCATTTACATGCTCCTGT
- NTRK2-R CATATTAGGAACCGGATCACCTG
- PTPN5-F ATGAGGGAGCCAGGAGTGAG
- PTPN5-R CACGGACAGAAAGACCAGCA
- PTPN7-F CCCAAGCAACTGGAAGAAGAAT
- PTPN7-R TTCCCGTCATAGCCTCGGAT

3.2 Sample Information

Exp. No.	Sample Name
1	1
2	2
3	3

3.3 qPCR Detection

3.3.1 Experimental Samples

cDNA samples were serially diluted 10-fold to produce 5 gradient points.

3.3.2 qPCR Reagents and Instrument

- Reagent: 2× SGExcel FastSYBR Mixture (B532955, Sangon)
- Instrument: LightCycler 480 II (Roche, Rotkreuz, Switzerland)

3.3.3 PCR Reaction Setup

1) Reaction mixture

Component	Concentration	Volume (µL)
SGExcel FastSYBR Mixture	2×	5
Forward primer (10 µM)	10 µM	0.2
Reverse primer (10 µM)	10 µM	0.2
RNase-free ddH_2O	—	3.6
Template (cDNA)	—	1
**Total**		**10**

2) Thermal cycling conditions

<table>
<tr><td rowspan="3">Thermal Cycler</td><td colspan="3">Times and Temperatures</td><td>Dissociation</td></tr>
<tr><td rowspan="2">Initial Steps</td><td colspan="2">Each of 45 cycles</td><td rowspan="4">Accoding to instrument guidelines</td></tr>
<tr><td>Melt</td><td>Anneal/Extend</td></tr>
<tr><td rowspan="2">LightCycler480 II</td><td>HOLD</td><td colspan="2">CYCLE</td></tr>
<tr><td>3 min 95°C</td><td>15 s 95°C</td><td>45 s 60°C</td></tr>
</table>

3) Instrument operation

After preparation, the loaded 384-well plate was placed into the LightCycler 480 II (Roche) for amplification.

4. Experimental Results

4.1 Standard Curve Results

1) Internal Reference Gene GAPDH (serial dilution)

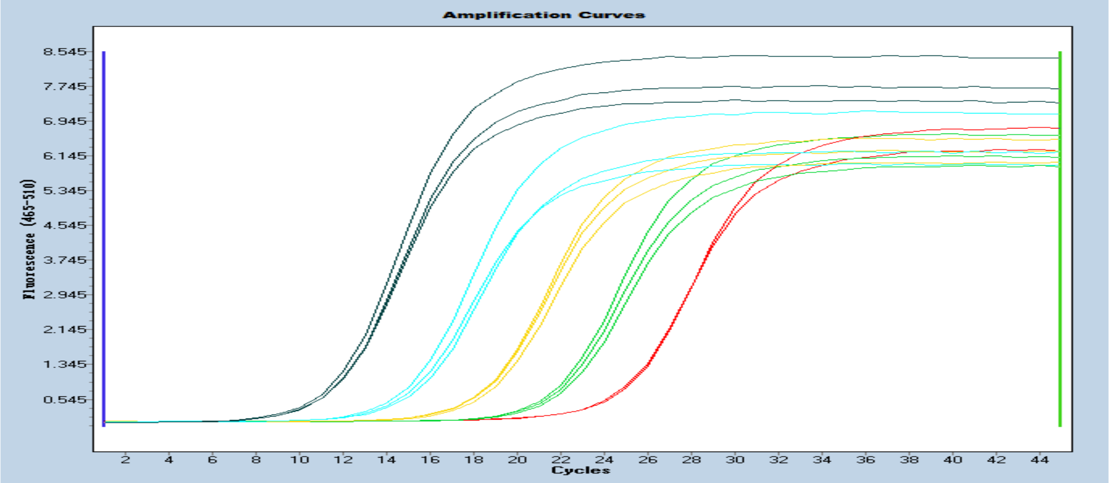

Five-point standard curve generated from 1×, 10×, 100×, 1000×, 10000× dilutions.

Melting curve

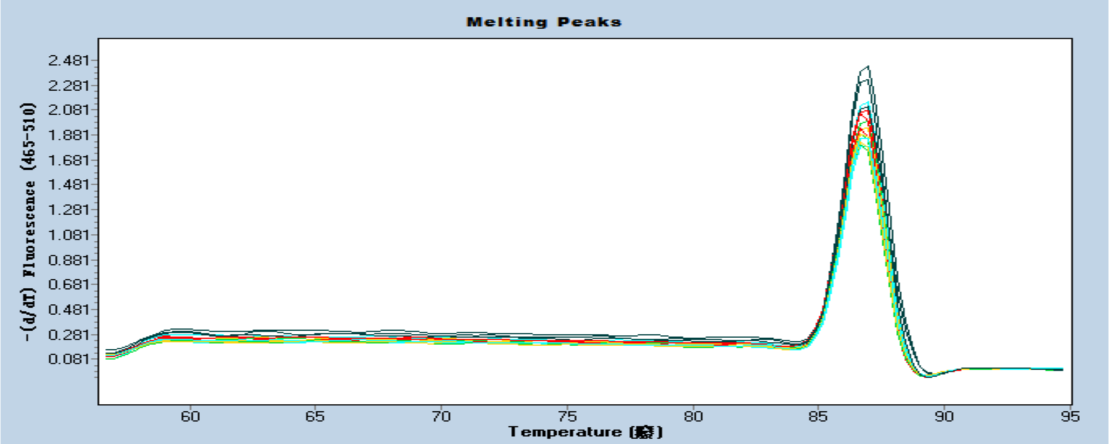

Standard curve

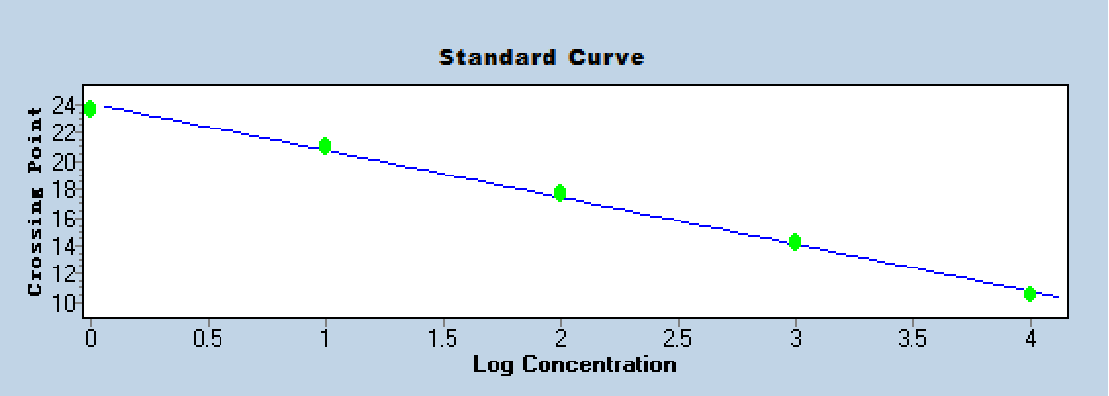

Note: Y-axis: Ct value; X-axis: LogCO (log_{10} copy number)

- Slope = −3.306
- Efficiency: $E=10^{-1/\ slope}-1=100.7\%$
- **Correlation coefficient:** $R^2 = 0.9954$
- Y-intercept = 24.03
- Error = 0.100

2) Target Gene CACNA1D

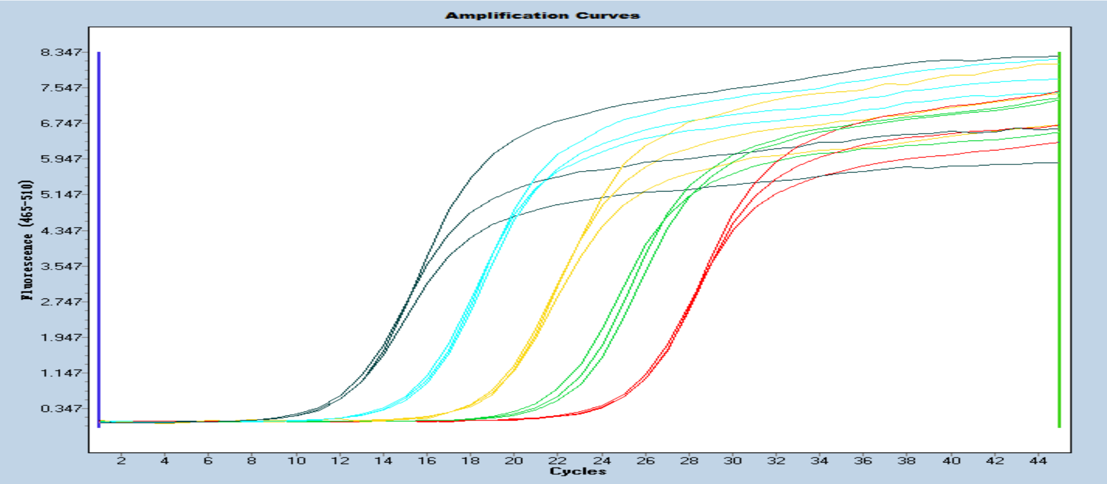

Five-point standard curve generated from 1×, 10×, 100×, 1000×, 10000× dilutions.

Melting curve

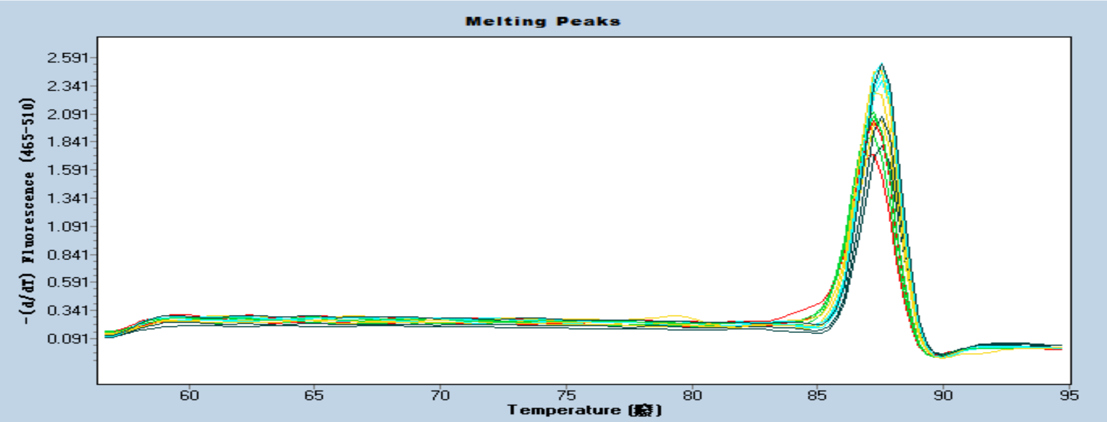

Standard curve

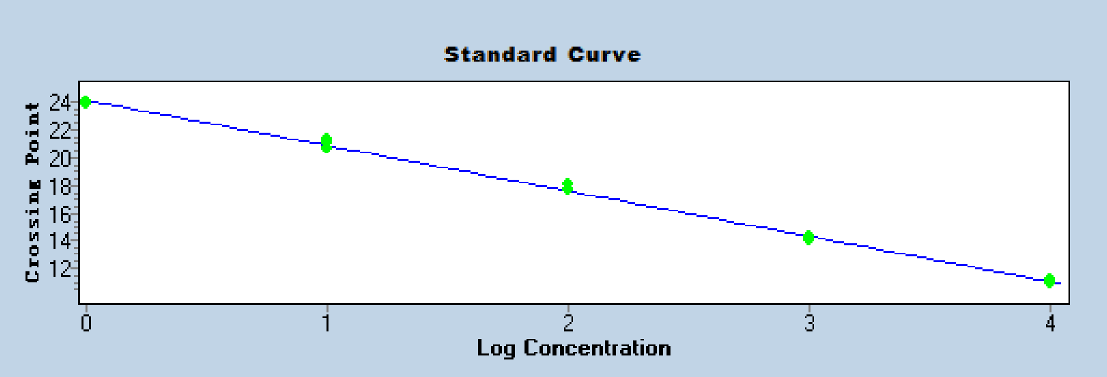

Note: Y-axis: Ct value; X-axis: LogCO (log_{10} copy number)

- Slope = −3.239
- Efficiency: $E=10^{-1/\text{slope}}-1$=103.6%
- **Correlation coefficient:** R^2 =0.9973
- Y-intercept = 24.09
- Error = 0.0710

3) Target Gene CACNA1H

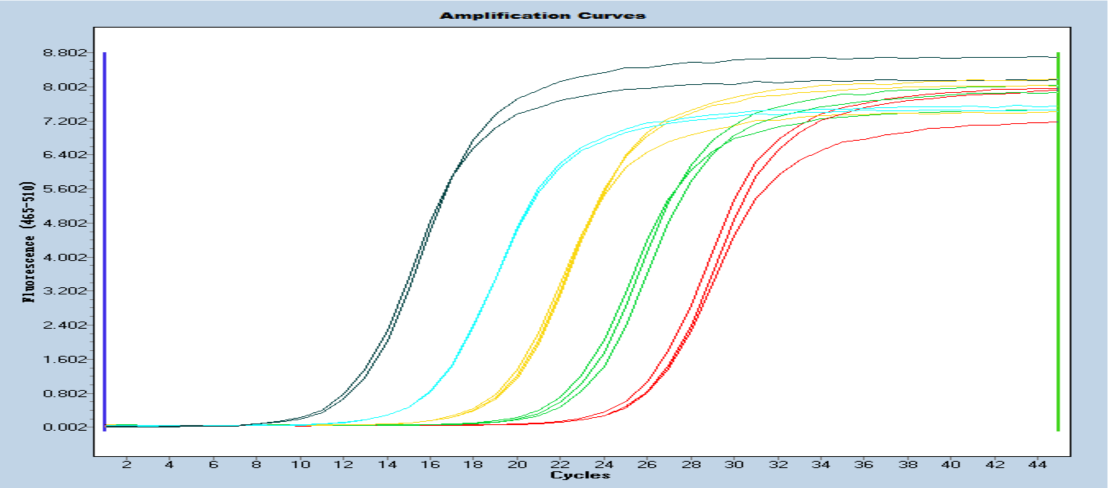

Five-point standard curve generated from 1×, 10×, 100×, 1000×, 10000× dilutions.

Melting curve

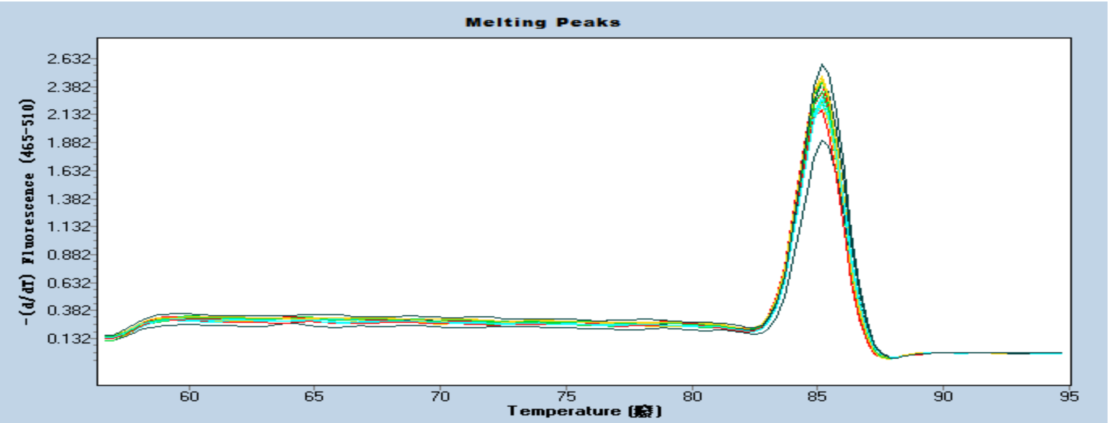

Standard curve

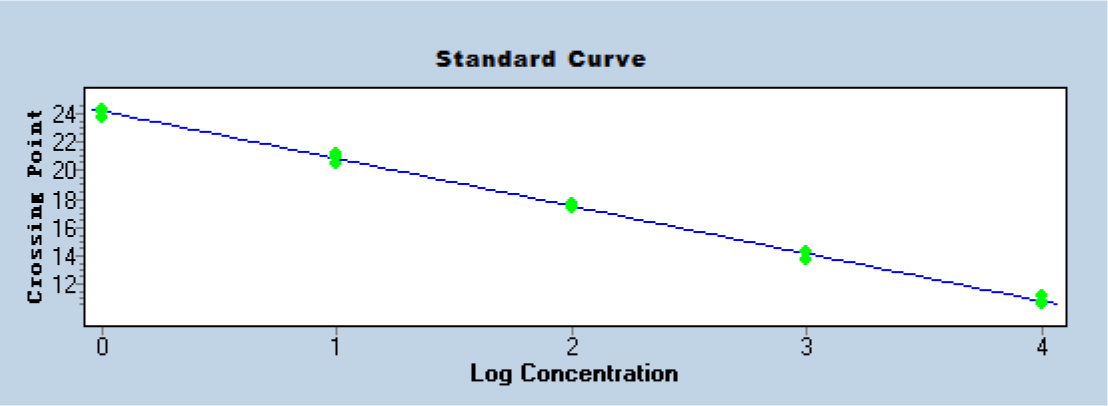

Note: Y-axis: Ct value; X-axis: LogCO (log_{10} copy number)

- Slope = −3.344
- Efficiency: $E=10^{-1/\ slope}-1$=99.1%
- **Correlation coefficient:** R^2 =0.9972
- Y-intercept = 24.19
- Error =0.0279

4) Target Gene MECOM

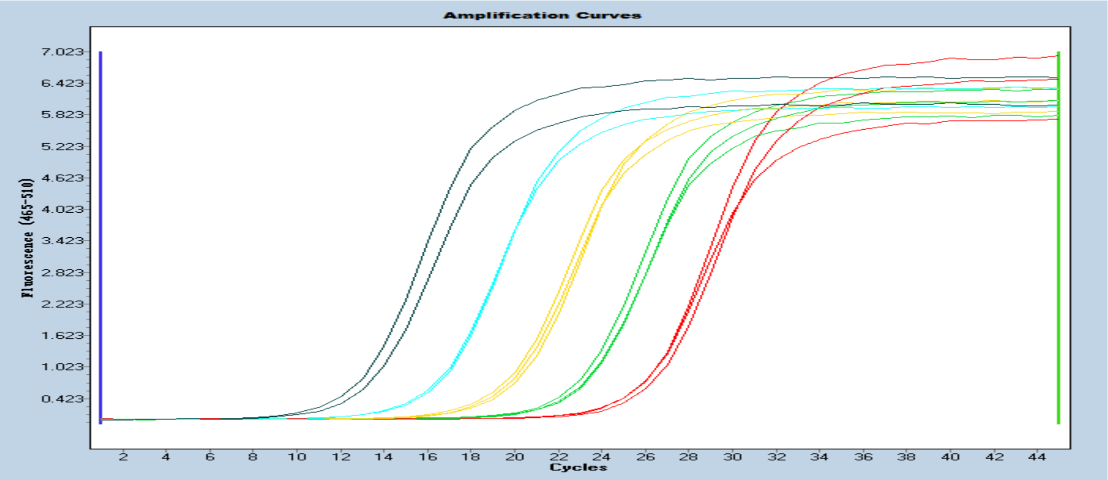

Five-point standard curve generated from 1×, 10×, 100×, 1000×, 10000× dilutions.
Melting curve

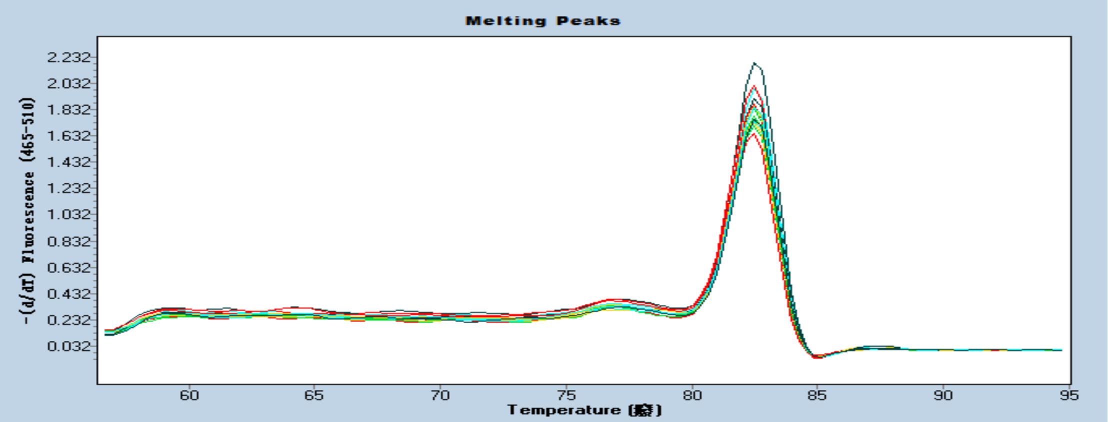

Standard curve

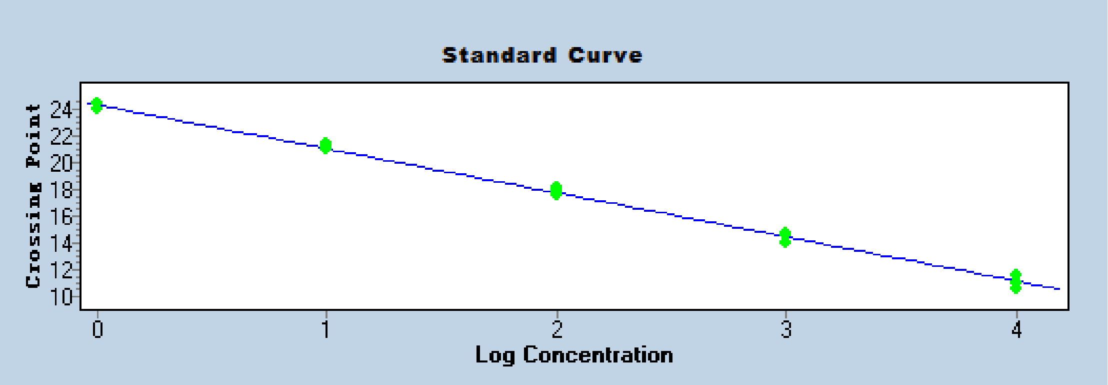

Note: Y-axis: Ct value; X-axis: LogCO ($\log_{10}$ copy number)

- Slope = −3.279
- Efficiency: $E=10^{-1/\text{slope}}-1$=101.8%
- **Correlation coefficient:** R^2 =0.9959
- Y-intercept = 24.29
- Error =0.0911

5) Target Gene NTRK2

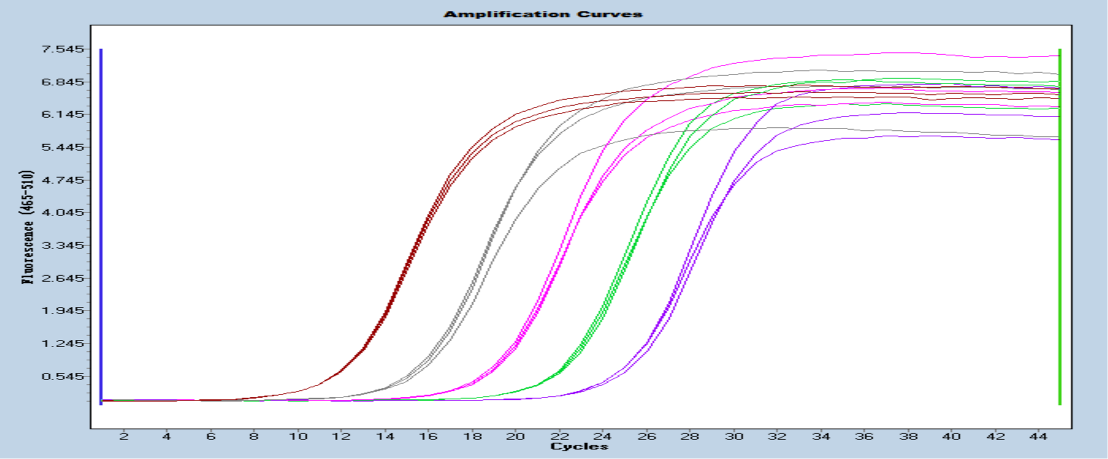

Five-point standard curve generated from 1×, 10×, 100×, 1000×, 10000× dilutions.

Melting curve

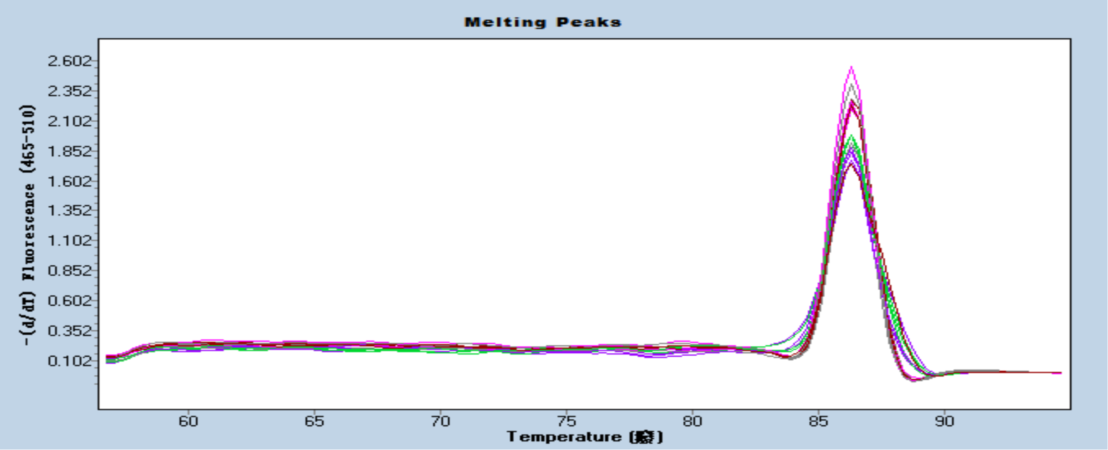

Standard curve

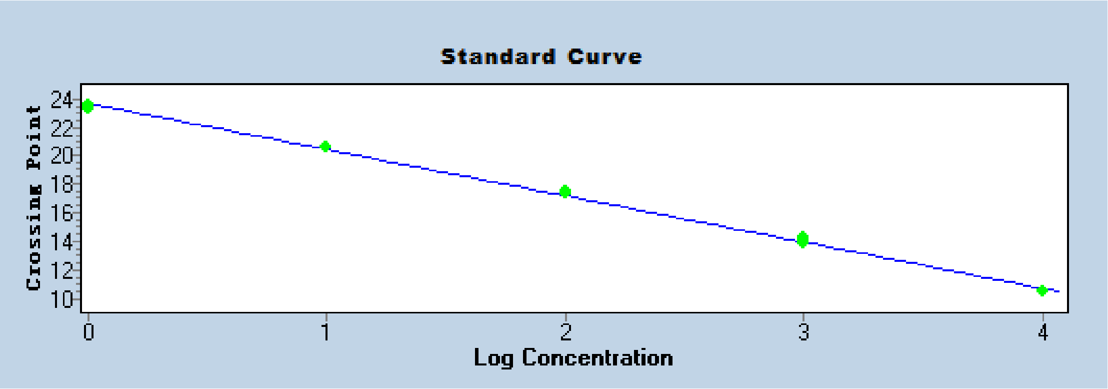

Note: Y-axis: Ct value; X-axis: LogCO (log_{10} copy number)

- Slope = −3.219
- Efficiency: $E=10^{-1/\ slope}-1=104.5\%$
- **Correlation coefficient:** R^2 =0.9978
- Y-intercept = 23.64
- Error =0.0918

6) Target Gene PTPN5

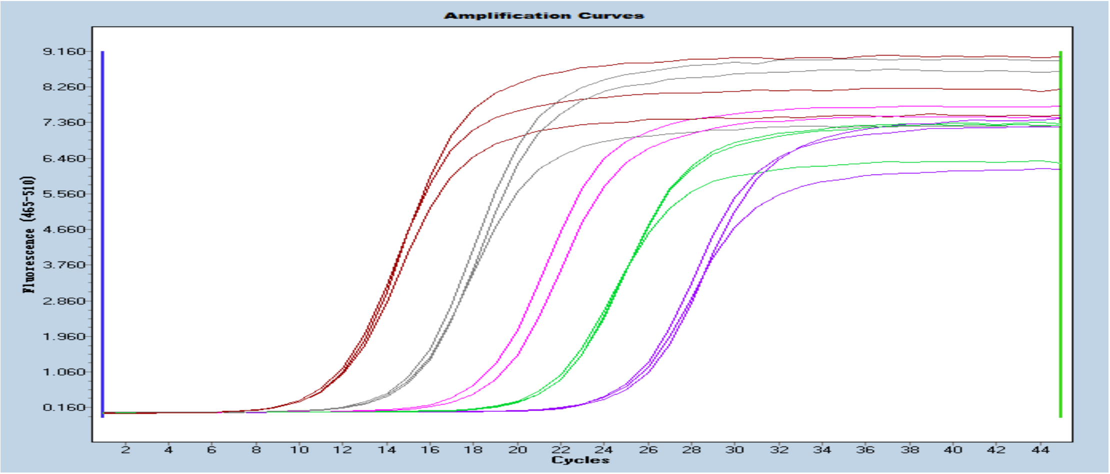

Five-point standard curve generated from 1×, 10×, 100×, 1000×, 10000× dilutions.

Melting curve

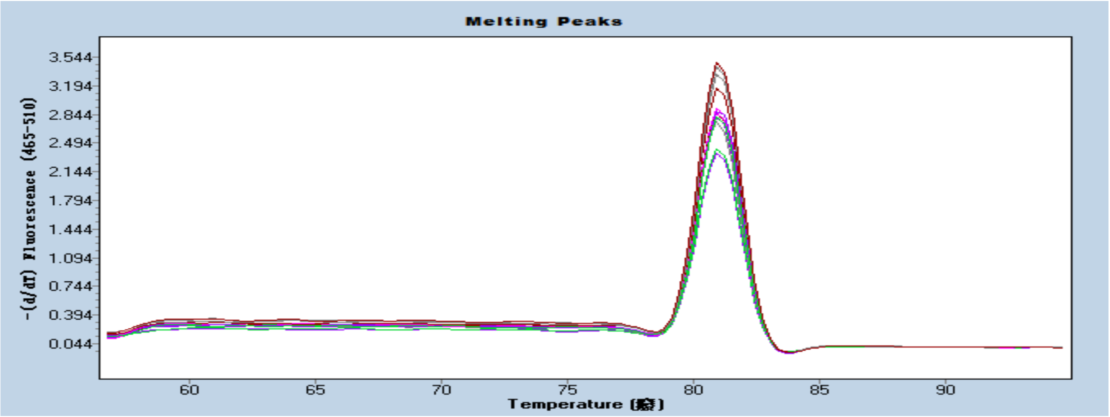

Standard curve

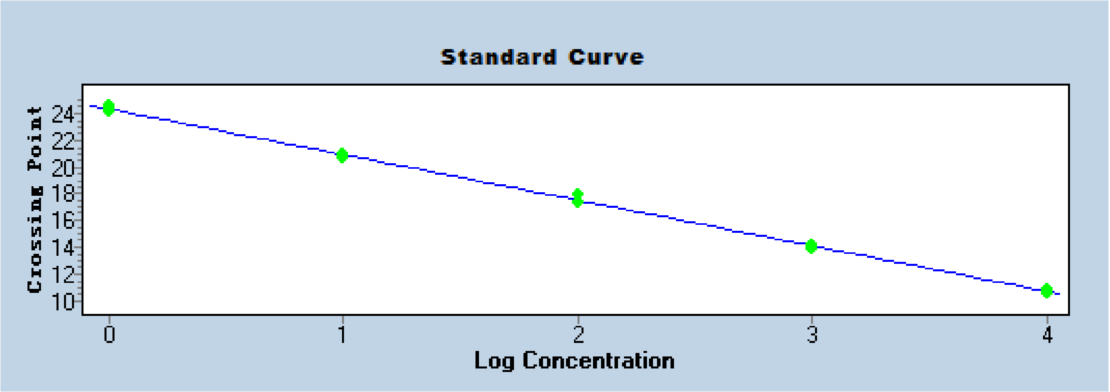

Note: Y-axis: Ct value; X-axis: LogCO (log_{10} copy number)

- Slope = −3.388
- Efficiency: $E=10^{-1/\ slope}-1=97.3\%$
- **Correlation coefficient:** R^2 =0.9986
- Y-intercept = 24.3
- Error =0.0336

7) Target Gene PTPN7

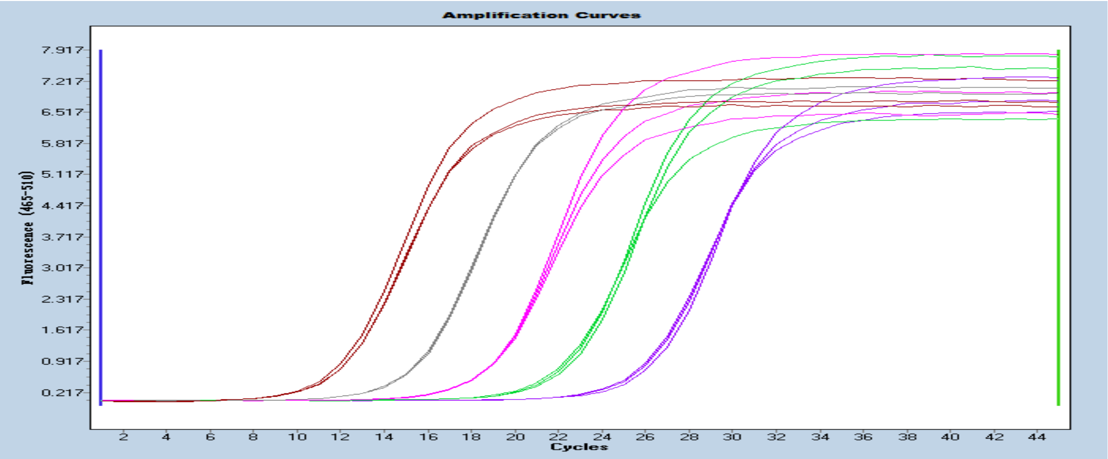

Five-point standard curve generated from 1×, 10×, 100×, 1000×, 10000× dilutions.

Melting curve

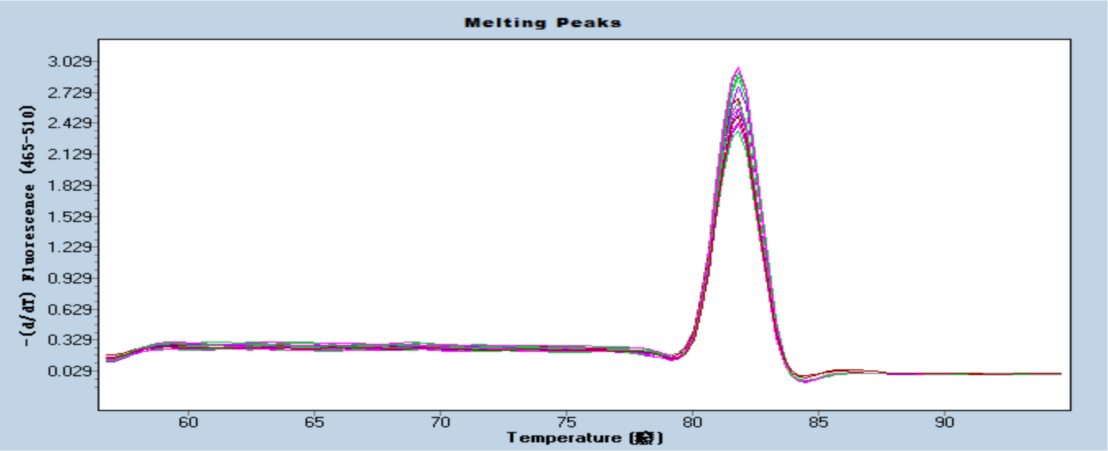

Standard curve

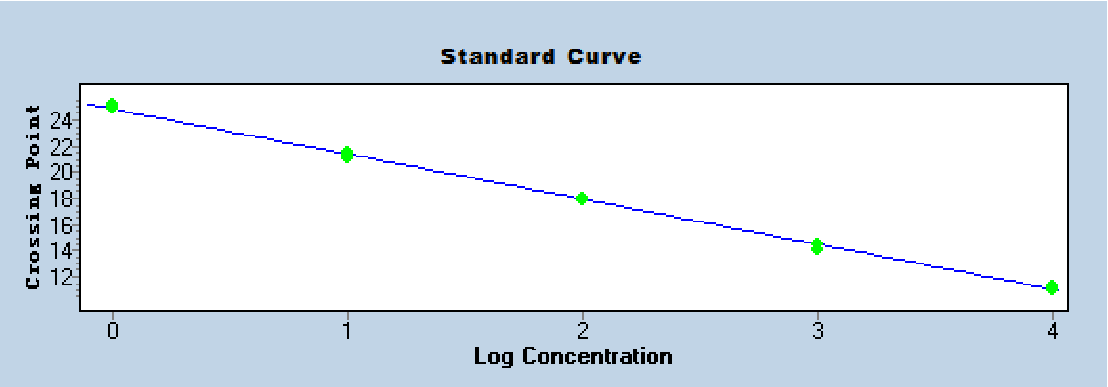

Note: Y-axis: Ct value; X-axis: LogCO (log_{10} copy number)

- Slope = −3.441
- Efficiency: $E=10^{-1/\text{slope}}-1$=95.3%
- **Correlation coefficient:** R^2 =0.9986
- Y-intercept = 24.81
- Error =0.0413
