# Supplementary material for: Overweight/Obesity and Hyperinsulinemia Impair Oocyte Developmental Competence via Cumulus Cell Dysfunction and Oxidative Stress-Mediated Follicular Microenvironment Remodeling
Source: Int J Mol Sci. 2026 Sep 16;27(18):8246. doi: 10.3390/ijms27188246 (PMC13607404; doi:10.3390/ijms27188246)
Supplement: Supplementary file 1 [file ijms-27-08246-s001.zip › Supplementary Material S3.pdf]

Shanghai OE Biotech Co., Ltd.

Sample Quality Control Report

Project Information			
**Project Name**	**Eukaryotic Reference Transcriptome Sequencing SA**	**Task Order No.**	**DZOE2025073598**
**Customer Name**	**Ting Wu**	**Project Contact**	**Ting Wu**
**Sample Type**	**Human-Granulosa Cell**		
**Sample Quantity**	**Ten**	**Order Date**	**2025-08-29**
**Lab Testing Personnel**	**OE1458,OE1646**	**Report Date**	**2025-09-02**

Sample Testing Method			
**Extraction Type**	**RNA**	**Extraction Method**	**RNA>>TRIzol® Reagent**
**Reagent Cat. No.**	**15596018**	**QC Method**	**NanoDrop, Agilent**

Sample Testing Results

No	Sample Name	Conc. (ng/μL)	A260/280	A260/230	Volume (μL)	Total Amount (μg)	28S/18S	RIN	Result
1	GC-24	13.7	1.96	1.45	20	0.27	1.7	9.2	B
2	GC-22	14.1	1.9	1.49	20	0.28	2.5	9.2	B
3	GC-53	39	1.83	1.88	20	0.78	1.9	10	A
4	GC-51	119.4	1.91	1.05	20	2.39	2.3	10	A
5	GC-52	25.2	1.89	1.56	20	0.5	1.4	9.6	A
6	GC-54	18.2	1.89	1.33	20	0.36	2.2	9.5	B
7	GC-83	16	1.82	1.65	20	0.32	2.2	9.4	B
8	GC-84	11.4	1.84	1.34	20	0.23	3	9.5	B
9	GC-91	22.5	1.84	1.27	10	0.23	2.4	9.2	B
10	GC-94	51.2	1.83	1.28	10	0.51	2	10	A

Sample Testing Profiles

【nt】 EL1(L) 1 2 3 4 5 6 7 8 9 10

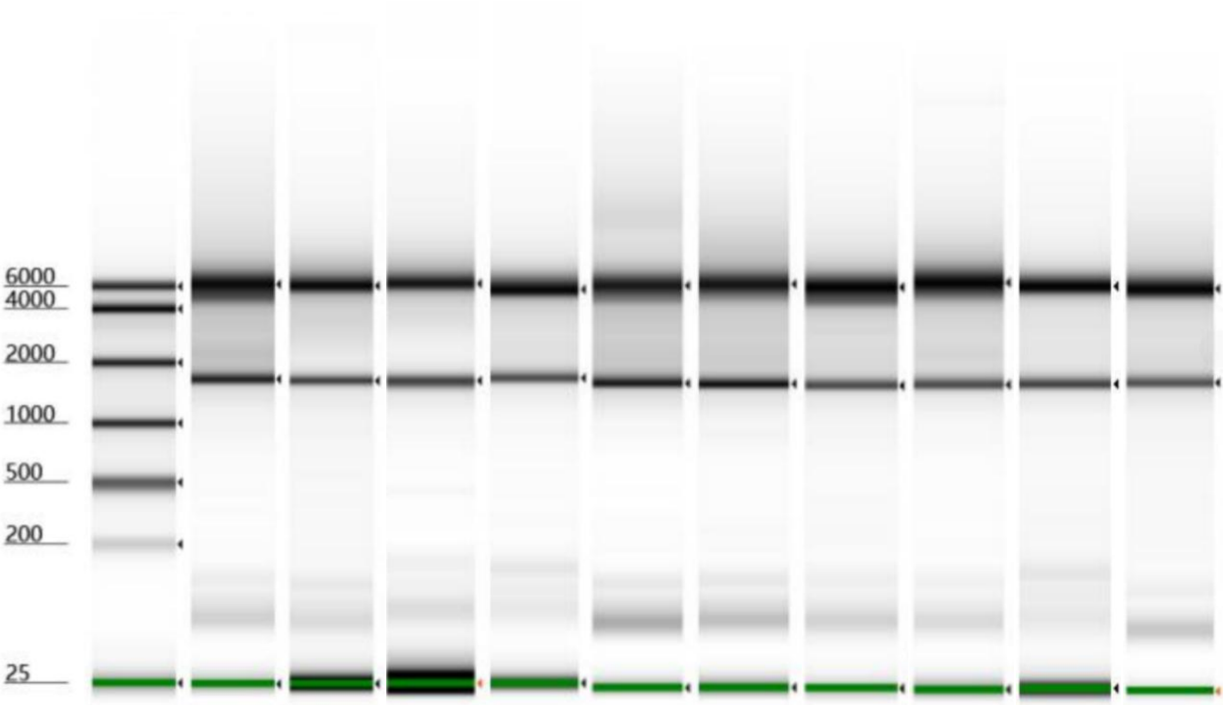

EL1:Electronic Ladder 1 2

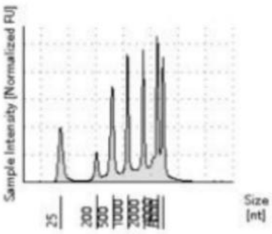

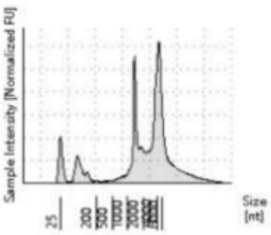

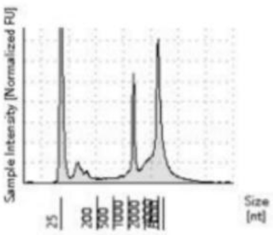

3 4 5 6

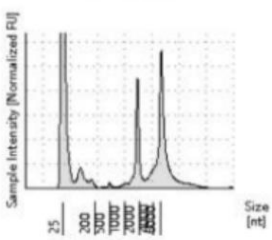

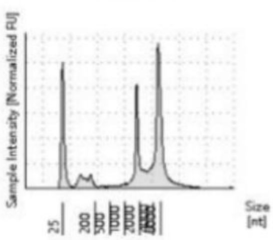

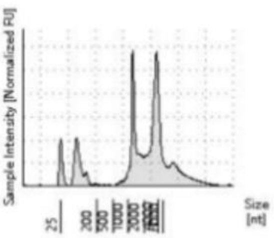

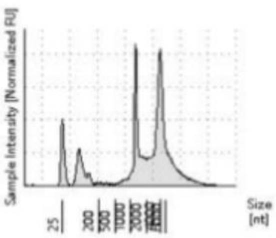

7 8 9 10

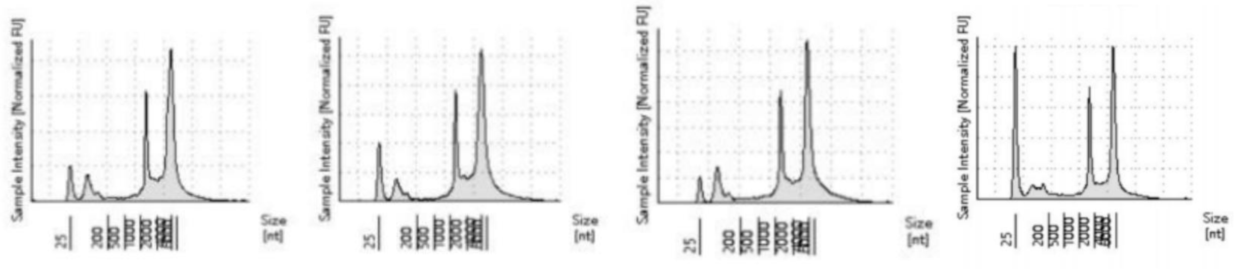
Sample Intensity [Normalized FU]
Size [nt]
25
200
500
1000
2000
